# Glucose Sensor MdHXK1 Phosphorylates and Stabilizes MdbHLH3 to Promote Anthocyanin Biosynthesis in Apple

**DOI:** 10.1371/journal.pgen.1006273

**Published:** 2016-08-25

**Authors:** Da-Gang Hu, Cui-Hui Sun, Quan-Yan Zhang, Jian-Ping An, Chun-Xiang You, Yu-Jin Hao

**Affiliations:** National Key Laboratory of Crop Biology, National Research Center for Apple Engineering and Technology, College of Horticulture Science and Engineering, Shandong Agricultural University, Tai-An, Shandong, China; Peking University, CHINA

## Abstract

Glucose induces anthocyanin accumulation in many plant species; however, the molecular mechanism involved in this process remains largely unknown. Here, we found that apple hexokinase MdHXK1, a glucose sensor, was involved in sensing exogenous glucose and regulating anthocyanin biosynthesis. *In vitro* and *in vivo* assays suggested that MdHXK1 interacted directly with and phosphorylated an anthocyanin-associated bHLH transcription factor (TF) MdbHLH3 at its Ser^361^ site in response to glucose. Furthermore, both the hexokinase_2 domain and signal peptide are crucial for the MdHXK1-mediated phosphorylation of MdbHLH3. Moreover, phosphorylation modification stabilized MdbHLH3 protein and enhanced its transcription of the anthocyanin biosynthesis genes, thereby increasing anthocyanin biosynthesis. Finally, a series of transgenic analyses in apple calli and fruits demonstrated that MdHXK1 controlled glucose-induced anthocyanin accumulation at least partially, if not completely, via regulating MdbHLH3. Overall, our findings provide new insights into the mechanism of the glucose sensor HXK1 modulation of anthocyanin accumulation, which occur by directly regulating the anthocyanin-related bHLH TFs in response to a glucose signal in plants.

## Author Summary

Glucose is considered as a major regulatory molecule in addition to being essential metabolic nutrients and structural components in higher plants. As is well known, hexokinase1 (HXK1) is a glucose sensor that integrates diverse signals to govern gene expression and plant growth in response to environmental cues. Previously, it is reported that the nuclear HXK1 forms a glucose signaling complex core with the vacuolar H^+^-ATPase B1 (VHA-B1) and the 19S regulatory particle of proteasome subunit (RPT5B), which influences the transcription of target genes. However, it is yet unknown if and how HXK1 directly targets TFs to modulate their function in the nucleus in plants. Our results reveal the important roles of MdHXK1 protein kinase in phosphorylating MdbHLH3 TF to modulate anthocyanins accumulation in response to glucose in apple.

## Introduction

In higher plants, sugars function as major regulatory molecules in addition to being essential metabolic nutrients and structural components. Sugars control gene expression to affect developmental and metabolic processes during the entire plant life cycle and function in response to biotic and abiotic stresses [1–3]. Therefore, rigorous sugar-sensing and sugar-signaling systems are critical for coordinating photosynthesis and carbon metabolism and for adapting to changes in environmental conditions to sustain normal plant growth and development.

Among the myriad of sugars in photosynthesis, glucose is the preferred carbon and energy source. Glucose is involved in many metabolic pathways, including the glycolytic process, in organisms ranging from unicellular microbes to plants and animals [4,5]. In addition to its metabolic function, glucose is the most intensively studied sugar molecule and functions in specific regulatory pathways to modulate plant growth and development [6,7]. Glucose signaling modulates the gene expression of enzymes in the glyoxylate cycle and photosynthesis pathway, and is also involved in the decision of whether to initiate the normal seedling establishment after seed germination [8,9].

Hexokinase 1 (HXK1) is the first plant sugar sensor identified [9,10]. The genetic evidence for HXK1 as a sugar sensor is the isolation of two *Arabidopsis gin2* (*glucose insensitive 2*) mutants, both of which are mapped to the *HXK1* gene [11]. In the *Arabidopsis* genome, there are three *HXKs* and three *HXK-like* (*HKLs*) genes, which execute a variety of physiological functions, including controlling subcellular localization, protein complex formation and tissue-specific expression patterns [12–14]. Moreover, five orthologous *HXKs* have been identified in the apple genome. Among them, MdHXK1, a well-known apple hexokinase, is highly homologous with *Arabidopsis* AtHXK1 [15]. Generally, HXKs are located on the outer mitochondrial membrane, plastids and even in the nucleus [13,14,16].

The regulatory role of HXK1 in sugar signaling has been identified and characterized in plants in the past two decades. In *Arabidopsis*, HXK1 forms a high-molecular-weight complex together with the V-ATPase subunit VHA-B1 and the proteasome 19S regulatory subunit RPT5B in the nucleus. This complex directly binds to the promoters of *CAB2* (*chlorophyll a/b binding protein 2*) and *CAB3* genes to confer glucose-mediated transcriptional regulation independent of glucose metabolism in the cytosol [17]. Both seedlings and adult plants of *vha-b1* and *rpt5b* mutants display similar phenotypes as the *gin2* mutant, demonstrating the crucial role of the interaction with HXK1 in glucose signaling [11,17]. In addition, glucose signaling mediated by HXK1 shows crosstalk with ABA, ethylene, auxin, cytokinin and brassinosteroid signaling [18–20]. However, whether HXK1-mediated signaling is involved in the regulation of anthocyanin biosynthesis in plants remains unclear.

Anthocyanins are ubiquitously present in various tissues and organs of plants, especially in the fruit, leaf and flower of ornamental crops. They are responsible for the red, purple and blue coloration of tissues and organs depending on the cellular conditions, such as pH value [21]. Colored organs, such as flowers and fruits, attract pollinators and seed-dispersing animals [22]. Anthocyanins are also antioxidant molecules that protect plants from damage by reactive oxygen species (ROS) [23–25]. These properties also make them interesting as food ingredients for human and animal nutrition. Anthocyanins are biosynthesized via the flavonoid pathway in the cytosol and are transported into the vacuole by vacuolar transporters, including ABC and MATE-type transporters [26,27].

The flavonoid biosynthetic pathway is transcriptionally controlled by a regulatory MYB-bHLH-WDR (WBM) complex containing WD-repeat proteins, basic helix-loop-helix bHLH and MYB transcription factors (TFs), which are highly conserved among higher plant species [28–31]. As the crucial components of the WBM complex, bHLH TFs promote anthocyanin biosynthesis by directly binding to the promoters of not only anthocyanin structural genes, such as *DFR* and *UFGT*, but also anthocyanin-associated *MYB* TF genes to activate their expression [31–34]. Interestingly, MdbHLH3 protein promotes anthocyanin accumulation partially through a putative phosphorylation modification in response to low temperature in apple [32]. However, the protein kinase that mediates the phosphorylation of MdbHLH3 protein is unknown.

Sugars induce anthocyanin biosynthesis in various plant species [35–37]. First, they provide carbon sources, skeletons and glucosides for anthocyanin biosynthesis [38,39]. Second, they increase the expression levels of biosynthetic structural genes and regulatory *MYB* genes [37,40]; however, the precise mechanism by which sugars modulate these genes remains unknown. The present study found that a protein kinase, MdHXK1, is involved in the regulation of anthocyanin biosynthesis in response to glucose by interacting with the phosphorylating and stabilizing MdbHLH3 protein. Subsequently, the function of MdHXK1 in the modulation of anthocyanin accumulation was characterized in apple calli and fruits. Finally, the potential application of HXK1-mediated glucose signaling in the genetic improvement of horticultural traits is discussed.

## Results

### MdHXK1 modulates anthocyanin accumulation mainly through glucose signaling, but not through the catalytic pathway, under the high-glucose condition

Previous studies have verified that glucose significantly induces anthocyanin biosynthesis in *Arabidopsis* seedlings [36]. Similarly, the effect of different concentrations of glucose (0–6%, w/v) and the HXK inhibitor glucosamine on anthocyanins accumulation was tested in *in vitro* shoot cultures of the ‘Gala’ apple cultivar. The results showed that glucose promotes anthocyanin accumulation in an HXK-dependent manner in apple (S1 Fig; S5 Text).

**Fig 1:**
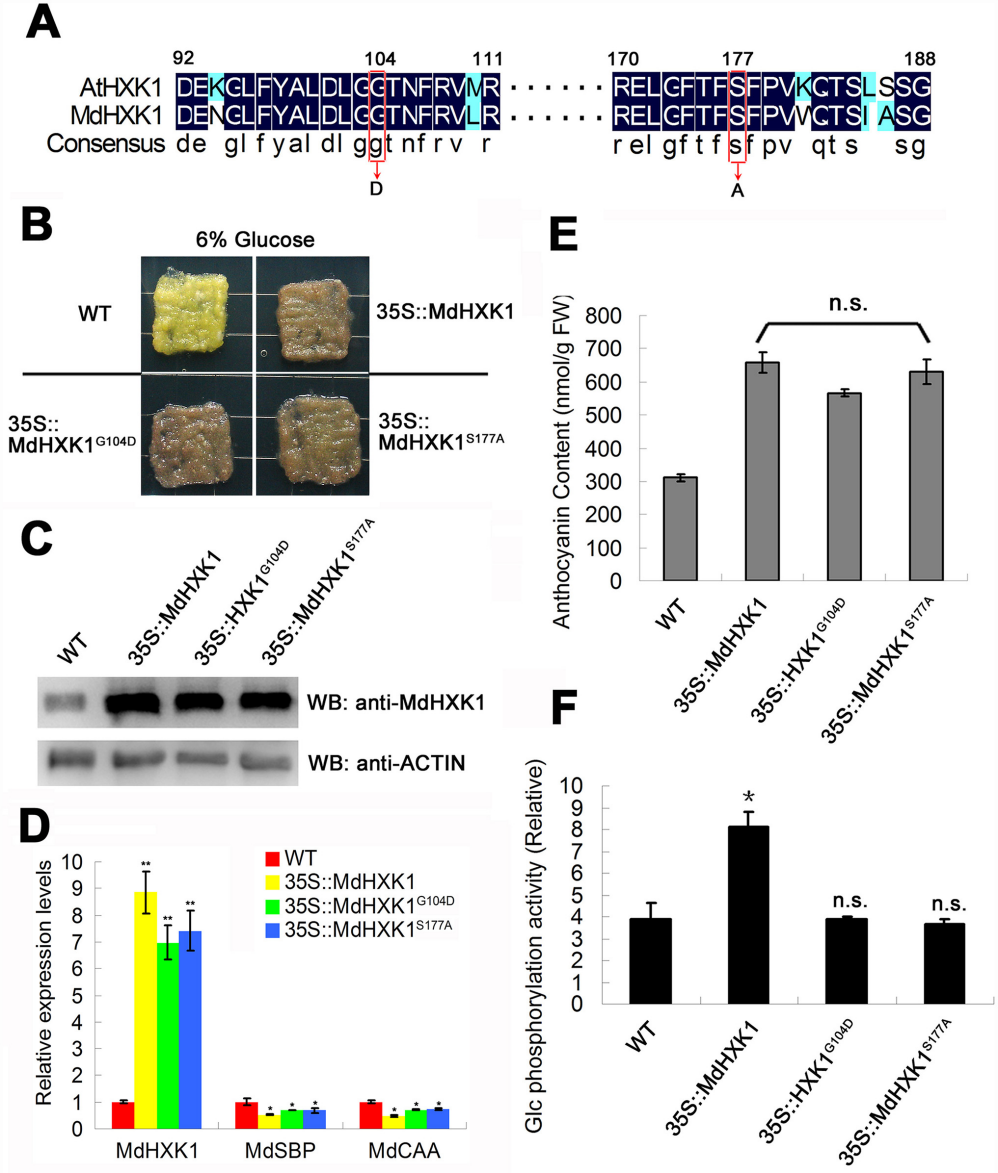
MdHXK1 modulates anthocyanin accumulation mainly through the glucose signaling pathway. **(A)** Partial amino acid sequences of HXK1 from *Arabidopsis* and apple orthologs are aligned. The highly conserved amino acids are highlighted with a black background. Conserved Gly (position 104 in AtHXK1 and MdHXK1) and Ser (position 177 in AtHXK1 and MdHXK1) residues are labeled with red boxes. **(B)** Phenotype of anthocyanin accumulation in the WT control and the *35S*::*MdHXK1*, *35S*::*MdHXK1*^*G104D*^ and *35S*::*MdHXK1*^*S177A*^ transgenic apple calli treated with 6% glucose. Note: Before being treated with 6% exogenous glucose, these apple calli were suffered from a dark (24 hours dark)-induced glucose starvation to deplete endogenous glucose. **(C)** Western blotting analysis of MdHXK1 protein abundance in the WT and transgenic apple calli. **(D)** *MdHXK1*-mediated glucose-dependent gene repression. *MdSBP*, sedoheptulose-biphosphatase (accession no. XM_008384867); *MdCAA*, carbonic anhydrase (accession no. XM_008387117). **(E)** and **(F)** Anthocyanin content **(E)** and glucose phosphorylation activity **(F)** in the WT and transgenic apple calli. The data are shown as the mean ± SE, which were analyzed based on more than 9 replicates. Statistical significance was determined using Student’s *t*-test in different apple calli lines. n.s., P > 0.01; *P < 0.01.

Because glucose controls anthocyanin accumulation in an HXK-dependent pathway in apple, it is reasonable to propose that this process is regulated by the catalytic or signaling function of HXK. We isolated the *MdHXK1* gene from apple to investigate this possibility. The predicted MdHXK1 protein is highly homologous with AtHXK1, which functions as not only a catalytically active kinase but also a glucose sensor in *Arabidopsis* [11,41]. Two catalytically inactive HXK1 mutants have been identified in *Arabidopsis*, namely, *HXK1*^*S177A*^ and *HXK1*^*G104D*^; these mutants retain signaling functions but not catalytic activities [11]. To investigate whether Ser and Gly at positions 177 and 104 of HXK1, respectively, are conserved between apple and *Arabidopsis*, an alignment of the amino acid sequence of MdHXK1 with AtHXK1 was performed. The result showed that apple MdHXK1 protein exhibited a high sequence similarity (77.31% identity) to *Arabidopsis* AtHXK1 (S2 Fig). The positions of 177 and 104 of apple MdHXK1 are the Ser and Gly residues, respectively, which were the same as those of *Arabidopsis* AtHXK1 (Fig 1A). These results suggest that the catalytically inactive apple MdHXK1 proteins MdHXK1^S177A^ and MdHXK1^G104D^ also exercised their functions in signaling but not catalytic activities, similar to *Arabidopsis*.

To rapidly determine whether the catalytically important G104 and S177 is involved in glucose-induced anthocyanin accumulation, two point mutations, i.e., MdHXK1^G104D^ and MdHXK1^S177A^, were made to the MdHXK1 protein. A total of three types of *35S*-driven vectors of *35S*::*MdHXK1*, *35S*::*MdHXK1*^*S177A*^ and *35S*::*MdHXK1*^*G104D*^ were generated and genetically transformed into apple calli of the 'Orin' cultivar (Fig 1B). Subsequently, these three transgenic apple calli and the wild-type (WT) control were used for immunoblotting assays with an anti-MdHXK1 antibody. The result demonstrated that the protein abundance of MdHXK1 was increased by 4.2-, 3.9- and 4.0-fold in the *35S*::*MdHXK1*, *35S*::*MdHXK1*^*G104D*^ and *35S*::*MdHXK1*^*S177A*^ transgenic apple calli, respectively, compared with the WT control (Fig 1C), indicating that the target genes were successfully transformed into and expressed in the transgenic apple calli. In addition, qPCR assays showed that *MdHXK1* repressed two classes of photosynthesis genes including *MdSBP* and *MdCAA* in *35S*::*MdHXK1*, *35S*::*MdHXK1*^*G104D*^ and *35S*::*MdHXK1*^*S177A*^ transgenic apple calli but not WT apple calli (Fig 1D), suggesting that *Gly104* and *Ser177* mutations have similar effect on MdHXK1 as *Arabidopsis* AtHXK1.

As a result, the three transgenic apple calli produced nearly the same levels of anthocyanins to each other but at a considerably higher level than in the WT control under 6% glucose conditions (Fig 1B and 1E), indicating that MdHXK1 and two point mutants successfully function to promote anthocyanin accumulation in these transgenic apple calli. In addition, the glucose phosphorylation activities were determined for the WT and these transgenic apple calli. The results showed that the *35S*::*MdHXK1* transgenic calli exhibited higher glucose phosphorylation activity than the *35S*::*MdHXK1*^*S177A*^ and *35S*::*MdHXK1*^*G104D*^ calli and the WT control (Fig 1F). However, there was no significant difference in glucose phosphorylation activities among the *35S*::*MdHXK1*^*S177A*^ and *35S*::*MdHXK1*^*G104D*^ transgenic calli and the WT control (Fig 1F). Collectively, these results suggest that MdHXK1 modulates anthocyanin accumulation mainly through glucose signaling, but not the catalytic pathway, under the high-glucose (6%) conditions.

Furthermore, the WT and aforementioned three transgenic calli were also treated with a low glucose concentration (1%) to induce anthocyanin accumulation. The result demonstrated that the *35S*::*MdHXK1*^*S177A*^ and *35S*::*MdHXK1*^*G104D*^ transgenic apple calli produced more anthocyanins than the WT controls but less anthocyanins than the *35S*::*MdHXK1* transgenic calli (S3A and S3B Fig). However, the glucose phosphorylation activities of the *35S*::*MdHXK1*^*G104D*^ and *35S*::*MdHXK1*^*S177A*^ transgenic apple calli showed no significant difference compared with the WT control but were considerably lower than the activities for the *35S*::*MdHXK1* transgenic calli (S3C Fig).

Taken together, these results indicate that MdHXK1 induces anthocyanin accumulation depending on both the catalytic activity and signaling under low-glucose conditions but mainly depending on signaling under high-glucose conditions.

### MdHXK1 interacts with MdbHLH3 via the conserved hexokinase_2 domain

To screen the target protein of MdHXK1 in its signal pathway, the *35S*::*MdHXK1-Myc* vector was constructed and genetically transformed into apple calli (S4A Fig). The *35S*::*MdHXK1-Myc* transgenic calli were used for co-immunoprecipitation (Co-IP) against the monoclonal anti-Myc antibody (S4B Fig). Subsequently, the Co-IPed proteins were analyzed with LC/MS to identify the potential proteins that interact with the MdHXK1 protein. The results showed that the anthocyanin-associated bHLH TF MdbHLH3 is a candidate (S1 Text).

**Fig 2:**
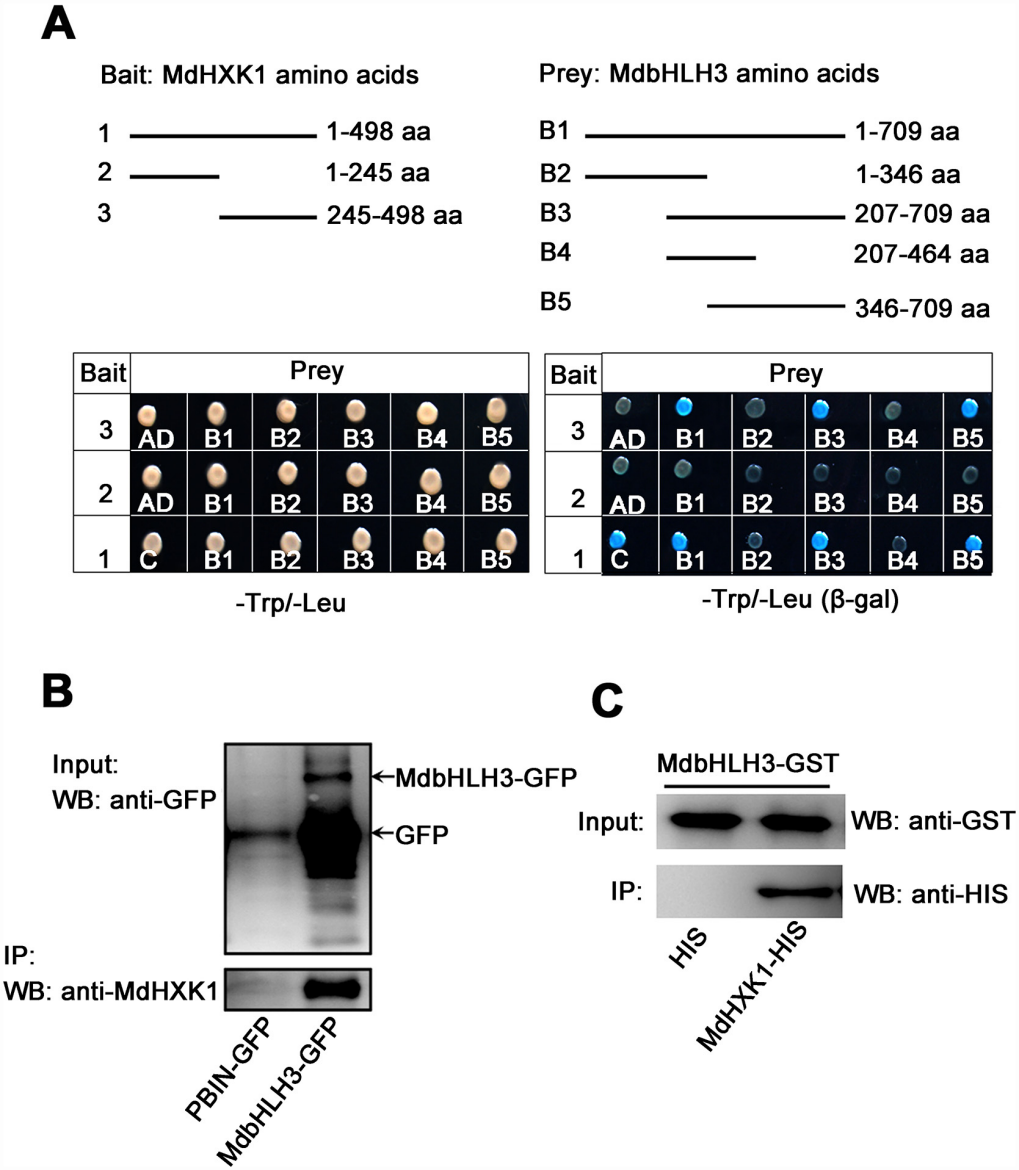
MdHXK1 physically interacts with MdbHLH3. **(A)** The C-terminus of MdHXK1 specifically interacts with the C-terminus of MdbHLH3 in a yeast two-hybrid assay. Top panels show the schematic representation of the different MdHXK1 and MdbHLH3 deletions in yeast vectors. Bottom panels show their interaction as indicated by yeast growth and β-gal staining in a serial of yeast two-hybrid assays. **(B)** *In vivo* Co-IP assays of the interaction between MdHXK1 and MdbHLH3 in transgenic apple calli. **(C)** *In vitro* GST pull-down assays of the interaction between MdHXK1 and MdbHLH3. Anti-His immunoblot (IB) shows the amount of MdHXK1-His bound by the indicated MdbHLH3-GST protein.

To determine whether MdHXK1 interacts with MdbHLH3 protein, yeast two-hybrid (Y2H) assays were performed. MdHXK1 protein contains two conserved hexokinase domains, i.e., hexokinase_1 and hexokinase_2 (S4C Fig). Therefore, the full-length cDNA of *MdHXK1* gene was divided into two fragments, i.e., MdHXK1^1-245aa^ and MdHXK1^245-498aa^. Subsequently, the full-length cDNA and two truncated mutants of the *MdHXK1* gene were inserted into the pGBT9 vector, independently, as the bait vectors. Moreover, the full-length *MdbHLH3* cDNA and its serially truncated mutants, as previously reported by Xie *et al*. [32], were inserted into the pGAD424 vector as the prey vectors. The different combinations of bait and prey vectors were transformed into yeast for Y2H assays. The results indicated that the full-length MdHXK1 strongly interacted with the full-length MdbHLH3 proteins. Furthermore, the truncated peptide MdbHLH3^346-709aa^, i.e., the C-terminus of MdbHLH3, interacted with MdHXK1 proteins at the hexokinase_2 domain MdHXK1^245-498aa^ but not at the hexokinase_1 domain MdHXK1^1-245aa^ (Fig 2A).

To further verify the interaction between MdHXK1 and MdbHLH3, an *in vivo* Co-IP assay using *35S*::*MdbHLH3-GFP* transgenic apple calli was conducted. The result indicated that the MdbHLH3-GFP fusion protein, but not the GFP negative control, interacted with MdHXK1 in apple calli (Fig 2B). In addition, a GST pull-down assay showed that a GST-tagged MdbHLH3 physically interacted with a His-tagged MdHXK1 *in vitro* (Fig 2C). These results indicate that the hexokinase_2 domain of MdHXK1 physically interacts with the C-terminus of the MdbHLH3 protein.

### Glucose induces phosphorylation of the MdbHLH3 protein at the Ser^361^ site

**Fig 3:**
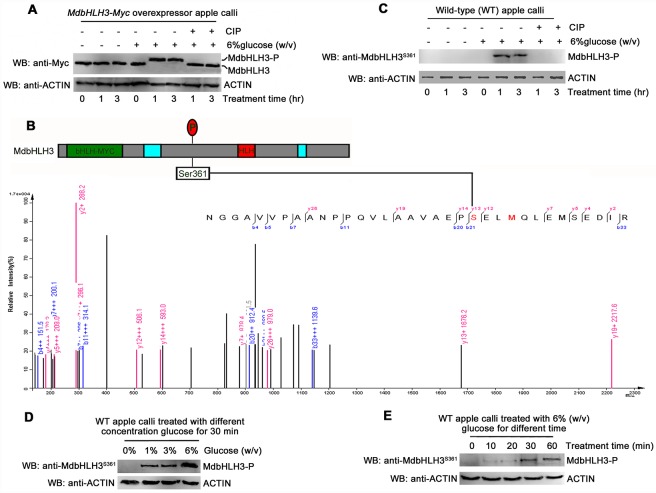
Glucose induces the phosphorylation of the MdbHLH3 protein at the Ser^361^ site. **(A)** Glucose induced the mobility shift of the MdbHLH3 protein, which was abolished by the phosphorylation inhibitor calf intestine alkaline phosphatase (CIP) in the *35S*::*MdbHLH3-Myc* transgenic apple calli. Note: MdbHLH3-P represents phosphorylated MdbHLH3 protein unless noted otherwise in this study. **(B)** Collision-induced dissociation mass spectrum showing the phosphorylation of Ser-361, a glucose-induced phosphorylation site in MdbHLH3. Top panel: the structural diagram of MdbHLH3 protein and its phosphorylation site. Bottom panel: the phosphorylation sites were identified using LC-MS/MS. MdbHLH3-Myc protein from transgenic apple calli was affinity purified as in (**A**) before being subjected to in-gel digestion with AspN. **(C)** Glucose induced the phosphorylation of the MdbHLH3 protein, which was abolished by CIP in WT apple calli. Western blotting was conducted with an anti-MdbHLH3^S361^ antibody specifically against the phosphorylation site. **(D)** and **(E)** The glucose-induced phosphorylation of MdbHLH3 protein depends on glucose concentration **(D)** and treatment time **(E)**. The WT apple calli was treated with different concentrations of glucose (0, 1%, 3% or 6%) for 30 min **(D)**, or treated with 6% glucose for different times (0, 10, 20, 30, or 60 min) **(E)**. A Western blotting assay was performed with an anti-MdbHLH3^S361^ antibody.

To examine how MdbHLH3 protein responds to glucose, an expression vector *35S*::*MdbHLH3-Myc* was constructed and genetically transformed into apple calli (S5A Fig). After treatment with or without 6% glucose, the *35S*::*MdbHLH3-Myc* overexpressing calli were used for Western blotting with the anti-Myc antibody. The results showed that the position of the MdbHLH3 proteins shifted from a faster- to a slower-migrating band in the transgenic apple calli treated with 6% glucose compared to those without glucose (Fig 3A), indicating that the MdbHLH3 protein was post-translationally modified in response to glucose. Furthermore, treatment with calf intestine alkaline phosphatase (CIP), which cleaves exposed phosphate residues from ribonucleotides and deoxyribonucleotides, converted the slower-migrating form of MdbHLH3 to the faster-migrating form (Fig 3A), indicating that the glucose-induced post-translational modification for the MdbHLH3 protein in apple calli is predominantly a phosphorylation.

To examine the potential phosphorylation sites of the MdbHLH3 protein, the glucose-induced phosphorylated MdbHLH3 protein in the *35S*::*MdbHLH3-Myc* overexpressing calli was captured with anti-Myc antibody-conjugated agarose beads and separated in an SDS-PAGE gel. After proteolytic digestion and purification, the protein sample was subjected to liquid chromatography-tandem mass spectrometry (LC-MS/MS) to detect the phosphorylation sites. The serine at residue 361 (Ser^361^) of the MdbHLH3 protein exhibited a high phosphopeptide signal intensity (Fig 3B; S2 and S4 Texts), suggesting that it is a potential phosphorylation site.

Subsequently, a monoclonal antibody specifically against the MdbHLH3 phosphorylation site at residue 361 was prepared and named as the anti-MdbHLH3^S361^ antibody (S5B Fig). This antibody specifically recognized the glucose-induced phosphorylation of MdbHLH3 protein in the WT apple calli (Fig 3C), which was consistent with the results shown in Fig 3A. These results indicate that glucose induces the phosphorylation of the MdbHLH3 protein at the Ser^361^ site in apple calli.

To examine whether the glucose concentration influences the phosphorylation of the MdbHLH3 protein, the WT apple calli were treated for 30 min with glucose concentrations of 0%, 1%, 3% and 6% and then used for immunoblotting with the anti-MdbHLH3^S361^ antibody. The results showed that the MdbHLH3 protein was not phosphorylated when the calli grew in absence of glucose, whereas the phosphorylation intensity of the MdbHLH3 protein increased gradually with glucose concentration (Fig 3D). Moreover, apple calli were treated with 6% glucose for different times (0, 10, 20, 30 and 60 min) to examine whether treatment time affects the phosphorylation of the MdbHLH3 protein. The results showed that the phosphorylation intensity of MdbHLH3 proteins in the calli gradually increased with the treatment duration (Fig 3E). These results indicate that the MdbHLH3 protein is phosphorylated in response to glucose and that this modification is positively associated with glucose concentration and treatment time.

In addition, glucose-induced phosphorylation of the MdbHLH3 protein could be observed in apple leaves (S5C Fig), indicating that the glucose-induced phosphorylation of the MdbHLH3 protein occurred in different apple tissues and organs.

### Glucose-induced MdbHLH3 phosphorylation is required for MdHXK1

**Fig 4:**
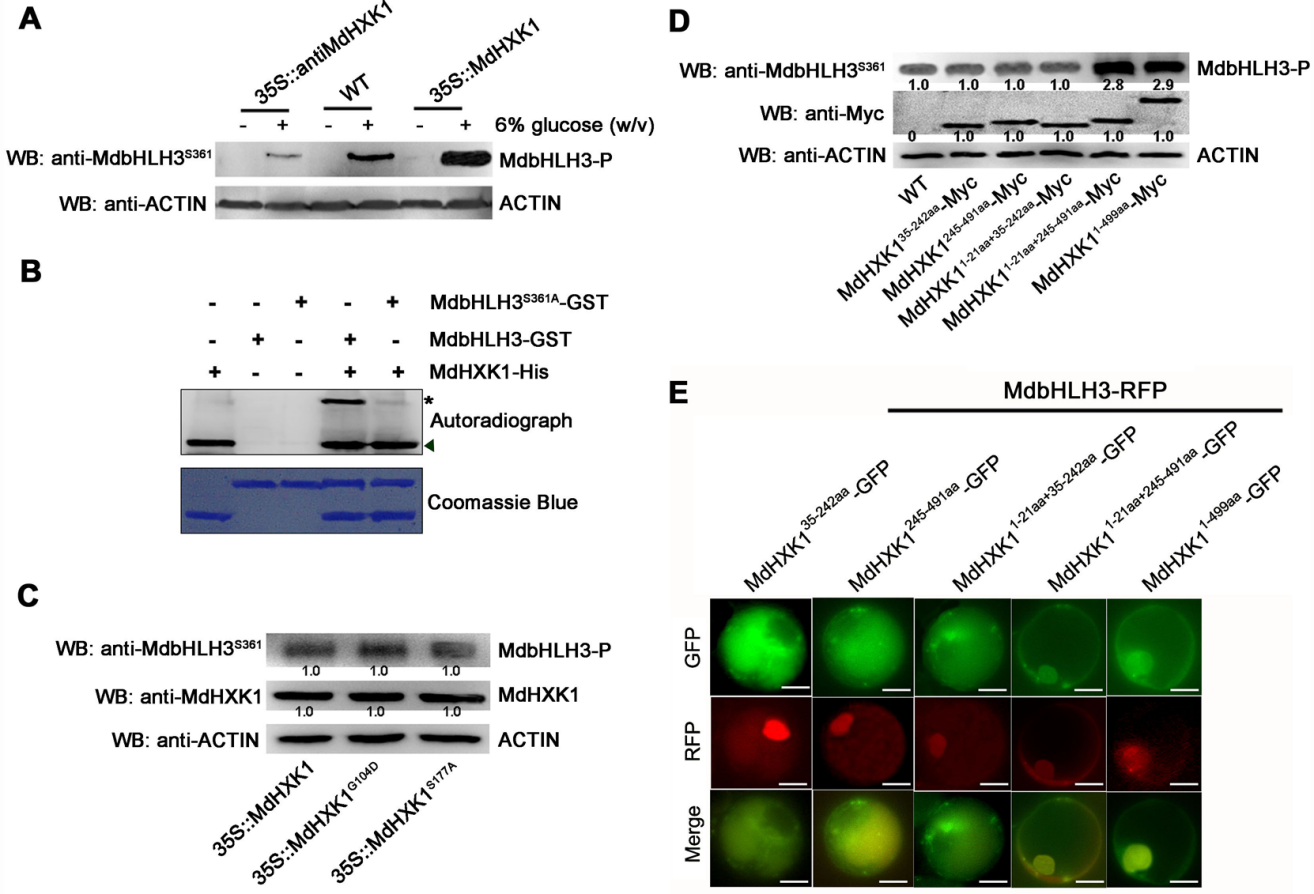
MdHXK1 mediates the glucose-induced phosphorylation of the MdbHLH3 protein. **(A)** The glucose-induced MdbHLH3 phosphorylation was enhanced in the *35S*::*MdHXK1* overexpressing apple calli but inhibited in the *35S*::*antiMdHXK1* suppressing apple calli. **(B)** MdHXK1 *in vitro* phosphorylates MdbHLH3 but not MdbHLH3^S361A^. The kinase assay was initiated by adding radiolabeled ATP to the mixture of MdHXK1-His kinase and MdbHLH3-GST (or MdbHLH3^S361A^-GST). SDS-PAGE gel with coomassie blue-stained MdHXK1-His, MdbHLH3-GST and MdbHLH3^S361A^-GST proteins (bottom panel); autoradiograph showing MdbHLH3 phosphorylation by MdHXK1 (top panel, top band labeled with asterisk) and MdHXK1 autophosphorylation (top panel, bottom bands labeled with triangle). **(C)** Mutation G104D or S177A of the MdHXK1 protein does not affect its ability to phosphorylate MdbHLH3. The *35S*::*MdHXK1*, *35S*::*MdHXK1*^*G104D*^ and *35S*::*MdHXK1*^*S177A*^ transgenic apple calli were used. Protein amounts were normalized based on the protein folds of the *35S*::*MdHXK1* transgenic apple calli. **(D)** Signal peptide and hexokinase_2 domain of MdHXK1 play a crucial role in the ability of MdHXK1 to phosphorylate the MdbHLH3 protein. The Myc-tag recombined vector plasmids of MdHXK1^35-242aa^ (hexokinase_1), MdHXK1^245-491aa^ (hexokinase_2), MdHXK1^1-21aa+35-242aa^ (Signal peptide + hexokinase_1), MdHXK1^1-21aa+245-491aa^ (Signal peptide + hexokinase_2) and MdHXK1^1-499aa^ (Signal peptide + hexokinase_1 + hexokinase_2) were transformed into the WT apple calli. Protein amounts were normalized based on the protein folds of the WT control. **(E)** Co-localization analysis of the full-length or truncated mutants of MdHXK1-GFP and MdbHLH3-RFP *in vivo*. The full-length and truncated mutants of MdHXK1 as mentioned in **(D)** were fused to the green fluorescent protein (GFP) tag. The full-length MdbHLH3 was fused to the red fluorescent protein (RFP) tag. For each image, two constructs, as indicated, were transferred into protoplasts of apple calli cells and then analyzed using confocal microscopy. Yellow colors in the merged images indicate the co-localization of the two signals. Bars = 20 μm. Note: In **(C)** and **(D)**, protein bands were quantified by scanning densitometry using a Hewlett Packard Scanjet scanner and Scanplot software. All of the protein amounts were normalized based on the protein folds of band 1.

Considering the interaction between MdHXK1 and MdbHLH3 proteins, it is reasonable to hypothesize that the MdHXK1 protein kinase mediates the phosphorylation of MdbHLH3 protein in apple calli. To verify this hypothesis, new transgenic apple calli, *35S*::*antiMdHXK1*, were obtained, which contained an antisense fragment specific to *MdHXK1* cDNA and exhibited considerably lower transcript and protein levels of MdHXK1 than the WT control (S6A and S6B Fig). Subsequently, immunoblotting assays with the anti-MdbHLH3^S361^ antibody were performed using the WT control and the *35S*::*MdHXK1* and *35S*::*antiMdHXK1* transgenic apple calli after treatment with or without glucose. The result showed that the *35S*::*MdHXK1* overexpressing calli exhibited a considerably higher phosphorylation level of the MdbHLH3 protein, whereas that of the *35S*::*antiMdHXK1*-suppressing calli were lower than the WT control in response to glucose treatment (Fig 4A). This result suggests that the MdHXK1 protein kinase is necessary, if not sufficient, for the glucose-induced phosphorylation of the MdbHLH3 protein in apple calli.

To further verify that MdHXK1 directly phosphorylates the MdbHLH3 protein, in-gel assays were conducted using prokaryon-expressed and purified MdHXK1-GST and MdbHLH3-His fusion proteins. As a result, MdbHLH3 protein was phosphorylated by the recombined MdHXK1 (Fig 4B). Furthermore, this *in vitro* phosphorylation assays were performed with anti-MdbHLH3^S361^ antibody. The result showed that MdbHLH3-His proteins were phosphorylated by MdHXK1, while MdbHLH3 mutation MdbHLH3^S361A^-His were not (S7 Fig). These results demonstrated that the MdbHLH3 protein is a direct substrate of the MdHXK1 protein kinase.

In addition, the phosphorylation status of the MdbHLH3 protein was determined in the glucose-treated *35S*::*MdHXK1*-, *35S*::*MdHXK1*^*G104D*^- and *35S*::*MdHXK1*^*S177A*^-overexpressing apple calli lines. Interestingly, there was no visible difference in the phosphorylation levels of these three transgenic calli (Fig 4C), indicating that the abolishment of MdHXK1 catalytic function as indicated by the phosphorylation activity is unable to affect the phosphorylation level of the MdbHLH3 protein.

### Both the hexokinase_2 domain and signal peptide are crucial for the MdHXK1-mediated phosphorylation of the MdbHLH3 protein

To further examine which kinase domain functions to phosphorylate the MdbHLH3 protein, vectors were constructed to contain the truncated *MdHXK1* cDNA fragments *MdHXK1*^*35-242aa*^ and *MdHXK1*^*245-491aa*^, which encode hexokinase_1 and hexokinase_2 domains, respectively. The resulting vectors *35S*::*MdHXK1*^*35-242aa*^*-Myc* and *35S*::*MdHXK1*^*245-491aa*^*-Myc* were genetically transformed into the WT apple calli, independently. Subsequently, the *35S*::*MdHXK1*^*35-242aa*^*-Myc* and *35S*::*MdHXK1*^*245-491aa*^*-Myc* transgenic apple calli were used for immunoblotting assays with anti-Myc and anti-MdbHLH3^S361^ antibodies, respectively. The results showed that the truncated proteins MdHXK1^35-242aa^ and MdHXK1^245-491aa^ were successfully expressed in the 2 transgenic calli. However, there was no visible difference in the phosphorylation level of MdbHLH3 between the WT control and 2 transgenic calli, i.e., *35S*::*MdHXK1*^*35-242aa*^*-Myc* and *35S*::*MdHXK1*^*245-491aa*^*-Myc* (Fig 4D).

In addition to the hexokinase_1 and hexokinase_2 domains, MdHXK1 also contains a signal peptide ranging from 1 to 22 amino acid residues at the N-terminus (S4C Fig). Given that signal peptides are polypeptide chains that are used as ‘address labels’ for sorting proteins to their correct subcellular destinations, it was hypothesized that the signal peptide of MdHXK1 is involved in the MdbHLH3 phosphorylation process. To verify this hypothesis, three vectors of MdHXK1 cDNA including the signal peptide domain, i.e., *35S*::*MdHXK1*^*1-21aa+35-242aa*^*-Myc*, *35S*::*MdHXK1*^*1-21aa+245-491aa*^*-Myc* and *35S*::*MdHXK1*^*1-499aa*^*-Myc*, were constructed and successfully transformed into the WT apple calli (Fig 4D). The resulting transgenic calli were used for immunoblotting assays with anti-Myc and anti-MdbHLH3^S361^ antibodies. The results showed that the phosphorylation intensities of MdbHLH3 proteins were considerably higher in the *35S*::*MdHXK1*^*1-21aa+245-491aa*^*-Myc* and *35S*::*MdHXK1*^*1-499aa*^*-Myc* transgenic calli than in the WT control. However, the level of MdbHLH3 phosphorylation was highly similar in the *35S*::*MdHXK1*^*1-21aa+35-242aa*^*-Myc* transgenic calli as in the WT control (Fig 4D). Therefore, the signal peptide and hexokinase_2 domain are crucial for MdHXK1-mediated phosphorylation of the MdbHLH3 protein.

To further verify the roles of the signal peptide and hexokinase_2 domain in the MdHXK1 protein on the MdbHLH3 phosphorylation process, a series of *35S* promoter-driven vectors that express fluorescence-tagged fusion proteins, including MdHXK1^35-242aa^-GFP, MdHXK1^245-491aa^-GFP, MdHXK1^1-21aa+35-242aa^-GFP, MdHXK1^1-21aa+245-491aa^-GFP, MdHXK1^1-499aa^-GFP and MdbHLH3-RFP, were constructed and used to determine their cellular distribution using an apple protoplast system. Upon co-transfection of the MdHXK1-related GFP fusion genes together with the MdbHLH3-RFP fusion gene into the apple protoplasts, the transformant protoplasts were observed in a subcellular localization assay using a laser confocal microscope. The results showed that MdHXK1^1-499aa^-GFP was co-localized with MdbHLH3-RFP in the nucleus (Fig 4E). Moreover, similar to MdHXK1^1-499aa^-GFP, MdHXK1^1-21aa+245-491aa^-GFP together with MdbHLH3-RFP resided in the nucleus, whereas other truncated peptides, including MdHXK1^35-242aa^-GFP, MdHXK1^245-491aa^-GFP and MdHXK1^1-21aa+35-242aa^-GFP, were not co-localized with MdbHLH3-RFP in the nucleus (Fig 4E).

Taken together, the signal peptide and hexokinase_2 domain of the MdHXK1 protein are essential for its nuclear co-localization together with the MdbHLH3 protein, which is crucial for MdHXK1-mediated phosphorylation of the MdbHLH3 protein.

### Phosphorylation modification stabilizes the MdbHLH3 protein and enhances its transcriptional activation of downstream genes

**Fig 5:**
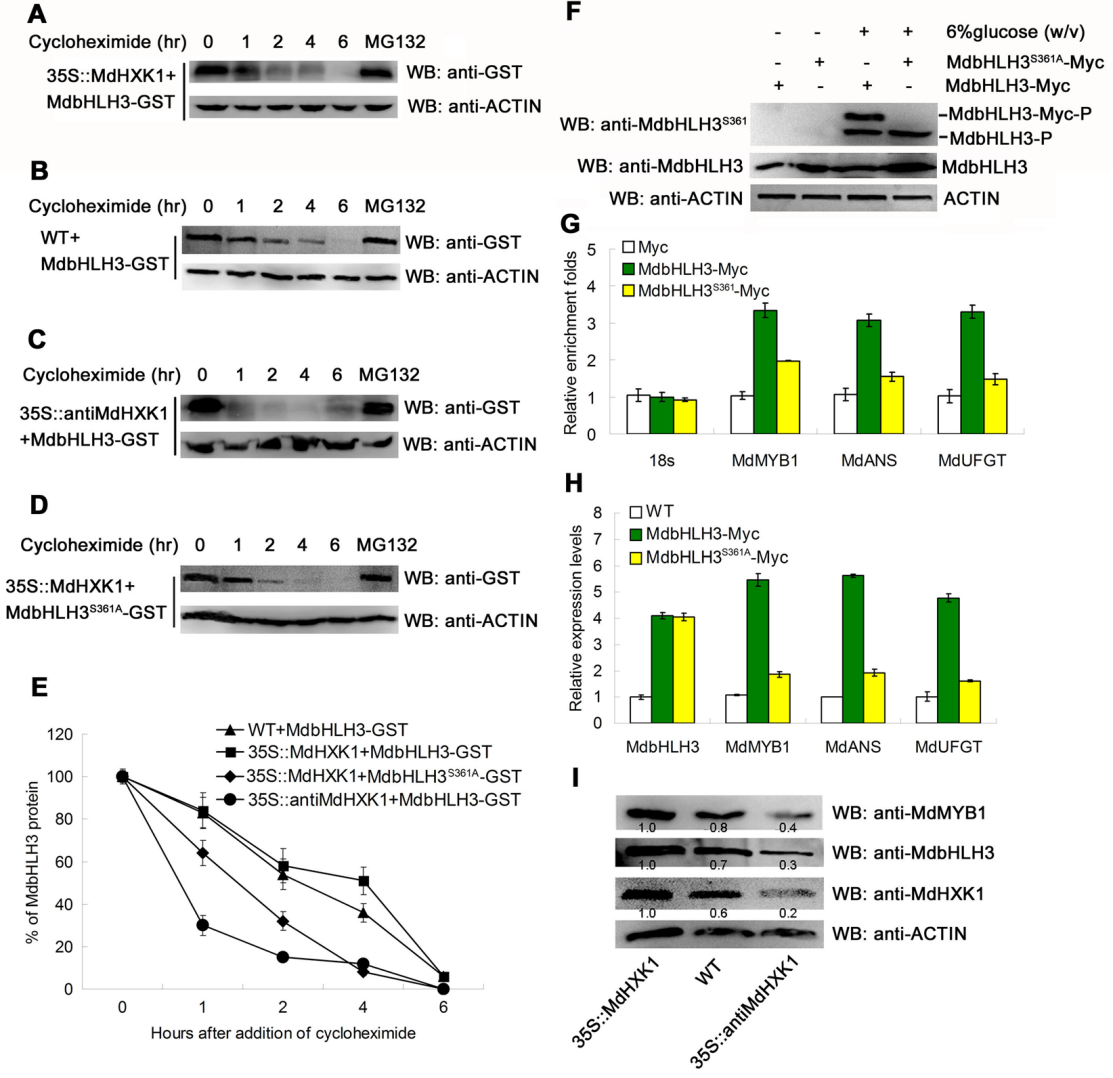
Phosphorylation modification stabilizes the MdbHLH3 protein and enhances its binding capacity to the promoters of downstream genes. **(A-C)** Cell-free degradation assays demonstrate that MdHXK1 stabilizes the MdbHLH3 protein. The recombined MdbHLH3-GST protein was co-incubated with the isolated total proteins extracted from the WT control (B) and the *35S*::*MdHXK1* **(A)** and *35S*::*antiMdHXK1* **(C)** transgenic apple calli. In addition, the mixed proteins were treated with 20 μg /mL cycloheximide for 0, 1, 2, 4 or 6 h. The degradation of MdbHLH3 protein was measured using Western blotting with an anti-GST antibody. MG132 was used as a positive control to stabilize the MdbHLH3 protein unless noted otherwise. **(D)** Ser361 is important for the MdHXK1-mediated stabilization of MdbHLH3. **(E)** The graph shows the quantitation of the Western blot data in **(A)**, **(B)**, **(C)** and **(D)**. **(F)** Western blotting assay of the phosphorylation intensity for MdbHLH3 and MdbHLH3^S361A^ proteins in *MdbHLH3-Myc* and *MdbHLH3*^*S361A*^*-Myc* transgenic calli. The anti-MdbHLH3^S361^ antibody was used. **(G)** ChIP-qPCR assays of the enrichments of the target gene promoters in the *35S*::*MdbHLH3-Myc* and *35S*::*MdbHLH3*^*S361A*^*-Myc* transgenic calli compared to the *35S*::*Myc* transgenic calli. **(H)** Relative expression levels of *MdMYB1*, *MdANS* and *MdUFGT* in the WT control and in the *35S*::*MdbHLH3-Myc* and *35S*::*MdbHLH3*^*S361A*^*-Myc* transgenic calli. **(I)** Protein abundance of MdHXK1, MdbHLH3 and MdMYB1 in the WT control and in the *35S*::*MdHXK1* and *35S*::*antiMdHXK1* transgenic apple calli. Protein amounts were normalized based on the protein folds of the *35S*::*MdHXK1* transgenic apple calli. In **(G)** and **(H)**, the data are shown as the mean ± SE, which were analyzed based on more than 9 replicates. Statistical significance was determined using Student’s *t*-test in different apple calli lines. n.s., P > 0.01; *P < 0.01; **P < 0 .001.

To examine whether MdHXK1 influences the stability of MdbHLH3 proteins, the prokaryon-expressed and purified MdbHLH3-GST fusion proteins were incubated with plant total proteins that were extracted from the WT control and the *35S*::*MdHXK1* and *35S*::*antiMdHXK1* transgenic apple calli. Subsequently, protein degradation assays were performed. The results showed that MdbHLH3-GST proteins were more stable in the protein extracts of the *35S*::*MdHXK1* transgenic calli than in those of the WT control (Fig 5A, 5B and 5E), whereas they were degraded at a more rapid speed in the protein extracts of *35S*::*antiMdHXK1* transgenic calli compared to those of the WT control (Fig 5C and 5E). These results suggest that MdHXK1-mediated phosphorylation of the MdbHLH3 protein may increase its stability.

To further verify that phosphorylation influences the stability of the MdbHLH3 protein, a site-directed S361A mutation was introduced into the MdbHLH3 protein. The mutated cDNA *MdbHLH3*^*S361A*^ was inserted into the expression vector for prokaryon-expression and purification of MdbHLH3^S361A^-GST fusion proteins, which were then incubated with the total proteins extracted from the WT calli. The protein sample was used for Western blotting with the anti-GST antibody. The results showed that the MdbHLH3^S361A^-GST proteins degraded at a rapid speed compared with the wild-type MdbHLH3-GST proteins (Figs 4B, 5D and 5E), indicating that the inhibition of phosphorylation promoted the degradation of MdbHLH3 proteins. In addition, MdHXK1 also enhanced the stability of the endogenous MdbHLH3 proteins (S8A–S8D Fig).

To examine whether phosphorylation of the MdbHLH3 protein influences its binding capacity to the downstream genes, such as *MdMYB1*, *MdANS* and *MdUFGT*, the *35S*::*MdbHLH3-Myc* and *35S*::*MdbHLH3*^*S361A*^*-Myc* transgenic apple calli were used for ChIP-PCR analysis (Fig 5F; S9 Fig). The results showed that the phosphorylated MdbHLH3-Myc protein exhibited a higher enrichment in the promoters of *MdMYB1* and anthocyanins biosynthetic structural genes than the non-phosphorylated MdbHLH3^S361A^-Myc (Fig 5G). As a result, those genes showed higher expression levels in the *35S*::*MdbHLH3-Myc* transgenic apple calli than the *MdbHLH3*^*S361A*^*-Myc* apple calli (Fig 5H). Furthermore, the abundance of the endogenous MdbHLH3 and MdMYB1 proteins were higher in *35S*::*MdHXK1* overexpressing calli but lower in *35S*::*antiMdHXK1* suppressing calli than in the WT control (Fig 5I).

Therefore, phosphorylation modification stabilizes the MdbHLH3 protein and enhances its transcriptional activation of downstream genes.

### MdHXK1 promotes anthocyanin accumulation in an MdbHLH3-dependent manner

**Fig 6:**
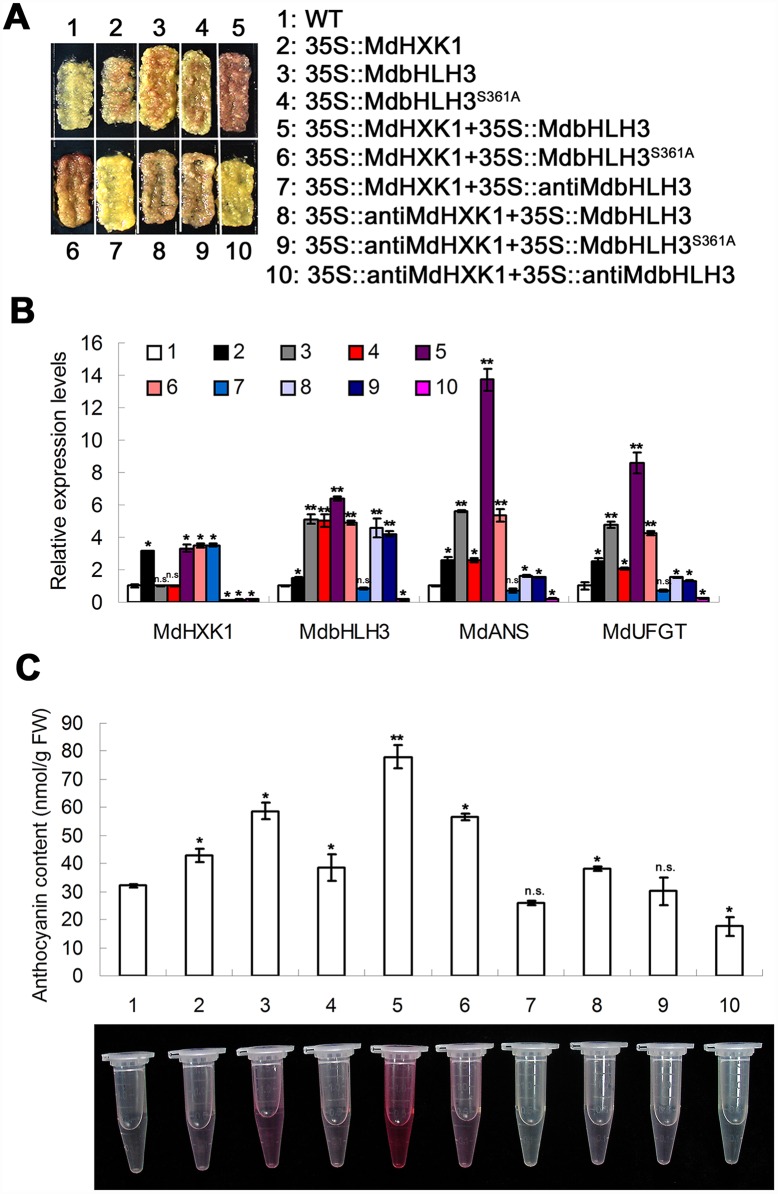
MdHXK1 controls anthocyanin accumulation via *MdbHLH3* in apple calli. **(A)** Anthocyanin accumulation in WT and transgenic apple calli grown on MS medium supplement with 6% glucose under 17°C plus UVB light. The numbers 1–10 represent the WT and transgenic apple calli containing different combinations of constructs, as indicated. **(B)** qRT-PCR analysis of the relative expression levels of *MdHXK1*, *MdbHLH3* and anthocyanin structural genes including *MdANS* and *MdUFGT* in the WT and transgenic apple calli. **(C)** Anthocyanin content of the WT and transgenic apple calli in **(A)**. In **(B)** and **(C)**, the data are shown as the mean ± SE, which were analyzed based on more than 9 replicates. Statistical significance was determined using Student’s *t*-test in different apple calli lines. n.s., P > 0.01; *P < 0.01; **P < 0 .001.

To examine whether and how MdHXK1 influences anthocyanin accumulation, the full-length sense ORFs and antisense cDNA fragments of *MdHXK1* and *MdbHLH3* (or *MdbHLH3*^*S361A*^) were inserted into the expression vectors downstream of *35S* promoters independently. The resulting vectors were then transformed into apple calli. In the present study, we obtained nine types of transgenic apple calli, namely, *35S*::*MdHXK1*, *35S*::*MdbHLH3*, *35S*::*MdbHLH3*^*S361A*^, *35S*::*MdHXK1*+*35S*::*MdbHLH3*, *35S*::*MdHXK1*+*35S*::*MdbHLH3*^*S361A*^, *35S*::*MdHXK1*+*35S*::*antiMdbHLH3*, *35S*::*antiMdHXK1*+*35S*::*MdbHLH3*, *35S*::*antiMdHXK1*+*35S*::*MdbHLH3*^*S361A*^ and *35S*::*antiMdHXK1*+*35S*::*antiMdbHLH3* (Fig 6A). The *MdHXK1* and *MdbHLH3* genes were successfully overexpressed or suppressed in the corresponding calli compared with the WT control (Fig 6B), indicating that the genetic transformation was successful in apple calli. As downstream genes, the transcript levels of *MdANS* and *MdUFGT* genes were positively correlated with that of the *MdbHLH3* gene; however, *MdANS* and *MdUFGT* were considerably lower in the *35S*::*MdbHLH3*^*S361A*^ transgenic calli than in the *35S*::*MdbHLH3* calli (Fig 6B). In addition, the transcription activity of the *MdANS* promoter was positively associated with the transcript level of *MdHXK1* genes (S10 Fig).

These transgenic apple calli were used to determine the anthocyanin content. The results showed that overexpression of *MdHXK1* and *MdbHLH3*, either alone or together, noticeably enhanced the anthocyanin content in the corresponding transgenic calli compared with the WT control (Fig 6C). Moreover, the *35S*::*MdbHLH3*^*S361A*^ transgenic calli produced less anthocyanins than the *35S*::*MdbHLH3* calli (Fig 6C), indicating that the phosphorylation site Ser361 is crucial for MdbHLH3 to regulate the biosynthesis of anthocyanins.

Furthermore, the *35S*::*MdHXK1* transgenic calli produced more anthocyanins, but the *35S*::*MdHXK1*+*35S*::*antiMdbHLH3* calli produced less than the WT control (Fig 6A and 6C), indicating that the suppression of the *MdbHLH3* gene inhibited the MdHXK1-mediated increase of anthocyanin biosynthesis. Therefore, MdHXK1 regulates anthocyanin accumulation at least partially, if not completely, depending on the presence of MdbHLH3.

### MdHXK1 works together with MdbHLH3 to modulate anthocyanin accumulation in apple fruits

**Fig 7:**
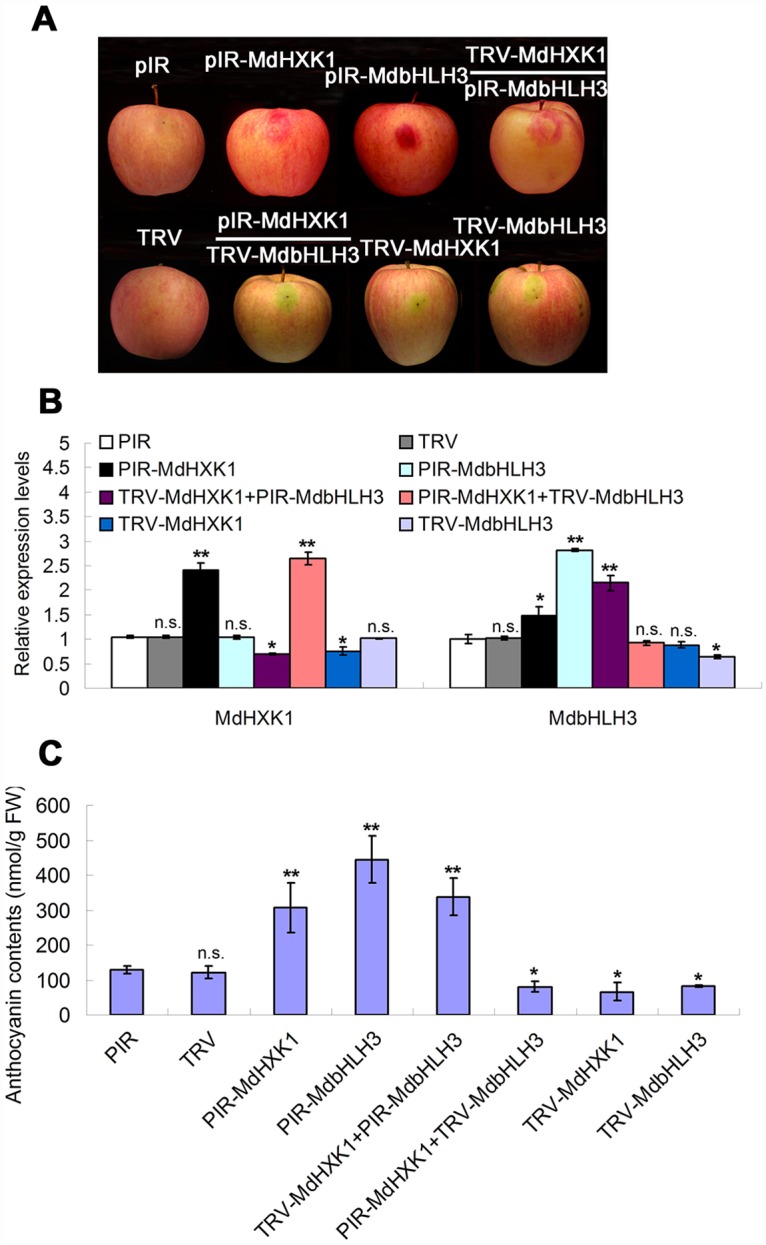
Transient expression of *MdHXK1* and *MdbHLH3* via the viral vector-based transformation alters anthocyanin levels in apple fruits. **(A)** Apple fruit peel coloration around the injection sites. The full-length cDNA of *MdHXK1* and *MdbHLH3* genes were cloned into the pIR vector for overexpression, whereas their antisense cDNA fragments were inserted into the TRV vector for suppression. The empty vectors were used as controls. **(B)** The qRT-PCR analysis of the relative expression levels of *MdHXK1* and *MdbHLH3* genes around the injection sites. **(C)** Anthocyanin content of the injected apple fruit peel in **(A)**. In **(B)** and **(C)**, the data are shown as the mean ± SE, which were analyzed based on more than 9 replicates. Statistical significance was determined using Student’s *t*-test in different apple calli lines. n.s., P > 0.01; *P < 0.01; **P < 0 .001.

To investigate whether MdHXK1 and MdbHLH3 regulate anthocyanin accumulation in apple fruits in a similar manner as in calli, a viral vector-based method was applied to alter their expression using vector pRI for overexpression and vector TRV for suppression. Four viral constructs, including pRI-MdHXK1, TRV-MdHXK1, pRI-MdbHLH3 and TRV-MdbHLH3, were obtained. Each construct and two combinations, i.e., TRV-MdHXK1+pRI-MdbHLH3 and pRI-MdHXK1+TRV-MdbHLH3, were used for fruit infiltration, with the empty vectors as controls (Fig 7A). The results showed that the transcript levels of *MdHXK1* and *MdbHLH3* genes were enhanced after being infiltrated with pRI-MdHXK1 and pRI-MdbHLH3 but decreased with TRV-MdHXK1 and TRV-MdbHLH3, respectively (Fig 7B).

Subsequently, anthocyanin levels were measured in apple peel tissues around the sites infiltrated with the different viral constructs. The results showed that both MdHXK1 and MdbHLH3 positively regulate anthocyanin accumulation and that the MdHXK1-mediated anthocyanin accumulation required MdbHLH3 in apple fruits (Fig 7C), similar to the apple calli (Fig 6C).

## Discussion

Sugar-induced anthocyanin accumulation is important for not only proper cell function [23,24] but also the quality formation of ornamental crops and fresh fruits [37,40,42]. Therefore, it is critical to elucidate the molecular mechanism underlying sugar-induced anthocyanin accumulation. The present study found that the glucose sensor MdHXK1, a hexokinase protein, stabilized the bHLH TF MdbHLH3 by phosphorylation modification, leading to an enhanced anthocyanin accumulation in apple.

### Glucose-induced anthocyanin accumulation results from MdHXK1-dependent glucose signaling and metabolic functions

Sugars are the major sources of carbon and energy metabolites and play key roles in plant growth and development. Sugars also act as effective signaling molecules throughout plant life [43,44]. In *Arabidopsis*, HXK1 is a crucial enzyme in glucose catabolism; HXK1 senses glucose and initiates its signaling pathway [11]. Glucose-promoted aliphatic glucosinolate biosynthesis is regulated by HXK1-mediated signaling via the MYB TFs MYB28 and MYB29 [45]. Most recently, it was reported that glucose treatment greatly enhances anthocyanin content and induces the expression of *PsWD40-2*, *PsMYB2*, *PsCHS1*, *PsCHI1* and *PsF3’H1* through glucose signaling in *Paeonia suffruticosa* cut flowers [37]. Among the WBM genes, *MYB* and *WD40* genes, but not *bHLH* genes, are induced at the transcriptional level by glucose. The present study found that a glucose-dependent signaling pathway is involved in the regulation of anthocyanin accumulation in apple. This process depended on functional MdHXK1, which directly phosphorylated and stabilized the WBM component MdbHLH3 protein at the post-translational level (Figs 6A–6C and 7A–7C). MdbHLH3 modulates both anthocyanin biosynthetic structural genes and the regulatory *MdMYB1* gene, thereby promoting anthocyanin accumulation [32].

Furthermore, anthocyanin accumulation is induced by glucose, which is not due to the osmotic effects of glucose (S1C and S1D Fig) [46]. MdHXK1 promoted anthocyanins accumulation mainly via the glucose signaling pathway under the high-glucose condition (Fig 1B–1F) and via both glucose metabolism and the signaling pathway under the low-glucose condition (S3 Fig). Given that sugars are the major sources of carbon and energy, higher plants require sugars for normal metabolism [9]. Glucose provides carbon skeletons for anthocyanin biosynthesis via its HXK1-dependent catalytic metabolism pathway, especially under low-glucose conditions (S3 Fig) [47]. Moreover, the MdHXK1-dependent glucose signaling pathway also plays a vital role in anthocyanin biosynthesis (Fig 1B–1E; S3 Fig). Therefore, glucose promotes anthocyanin biosynthesis depending on both signaling and metabolism under low-glucose conditions in apple. However, when carbohydrates are derived from glucose to meet the needs of anthocyanin synthesis under high-glucose conditions (e.g., 6% glucose), the glucose mainly served as signaling molecules to initiate anthocyanin biosynthesis (Fig 1B–1E). Taken together, glucose-induced anthocyanin accumulation is the result of MdHXK1-dependent glucose signaling together with catalytic metabolism pathways.

In the present study, the catalytic and signaling functions of MdHXK1 were characterized using its two catalytically inactive mutants, *MdHXK1*^*S177A*^ and *MdHXK1*^*G104D*^, in apple (Fig 1), both of which retain signaling functions but not catalytic activities, similar to their activities in *Arabidopsis* [11]. Most recently, Feng *et al*. [48] successfully resolved the crystal structures of two AtHXK1 inactive forms, AtHXK1^S177A^ and AtHXK1^G104D^, and analyzed the biochemical properties of AtHXK1 in *Arabidopsis*. These findings provide biochemical and structural insights into how HXK1 functions at the atomic level, thereby providing a structural explanation for the dual functions of HXK1 in plants.

### The nuclear HXK1 complex regulates glucose-mediated transcription activation

As the most important glucose sensor, HXK1 is involved in diverse signaling functions, particularly in the regulation of gene expression. In plants, HXK1 is mainly localized in the cytosol; however, a minor degree of HXK1 is also present in the nucleus [49]. In the nucleus, this minor portion of HXK1 interacts with the B1 subunit of the V-ATPase (VHA-B1) and with a 19S regulatory particle of the proteasome subunit (RPTB5), leading to the formation of unexpected nuclear HXK1 complexes [49]. A large number of putative TFs identified in the nuclear HXK1 complexes interact directly with VHA-B1 and/or RPT5B but not directly with HXK1 [49]. In addition, nuclear-localized HXK1 has also been implicated in the control of the transcriptional activity and proteasome-mediated degradation of EIN3 (ethylene-insensitive3), a key transcriptional regulator in ethylene signaling [50]. The present study found that HXK1 directly interacted with MdbHLH3 (Fig 2B–2D), a key bHLH transcriptional regulator in anthocyanin biosynthesis [32]. However, it is unclear whether the HXK1/VHA-B1/RPT5B nuclear complex is also involved in these processes.

Furthermore, a R2R3 MYB regulator MdMYB1 interacts with the N-terminus of MdbHLH3 to regulate anthocyanin biosynthesis [32]. The present study found that the hexokinase_2 domain of MdHXK1 strongly interacted with the C-terminus of MdbHLH3 to modulate anthocyanin accumulation (Fig 2B). Therefore, there is no competition for the interaction of the MdbHLH3 protein with MdMYB1 and MdHXK1 in the regulation of anthocyanin biosynthesis.

### MdbHLH3 phosphorylation may be an MdHXK1-mediated single-site phosphorylation event

In apples, a putative phosphorylation modification is involved in the MdbHLH3-mediated anthocyanin accumulation in response to low temperature [32]. However, the protein kinase that mediates the phosphorylation of MdbHLH3 protein is not yet identified. The present study found that the MdHXK1 protein kinase is directly involved in the glucose-induced phosphorylation of MdbHLH3 protein, thereby modulating anthocyanin biosynthesis (Fig 4A and 4B). In addition, the hexokinase_2 domain of MdHXK1, which may be required for signal peptide cleavage based on its functions in protein secretion and subcellular localization [51,52], plays a key role in the phosphorylation of the MdbHLH3 protein (Fig 4D and 4E).

Additionally, several bHLH TFs are phosphorylated by external environmental stimuli. For example, multiple light-induced Ser/Thr phosphorylation sites are found in the phyB-interacting bHLH TF PIF3 in *Arabidopsis* [53]. Multisite light-induced phosphorylation of the bHLH TFs PIF1 and PIF5 has been confirmed using photobiological and genetic approaches [54,55]. In addition to PIFs, another bHLH TF, TWIST1, is phosphorylated at Thr125 and Ser127 to control pro-metastatic functions in prostate cancer cells [56]. In contrast to the aforementioned bHLH TFs, the bHLH TF speechless is phosphorylated to promote stomatal development at a single serine 186 site in *Arabidopsis* [57]. Similarly to the bHLH TF speechless, only a single phosphorylation site in the bHLH TF MdbHLH3 protein was detected in apple (Fig 3B and 3C; S2 Text), suggesting that MdbHLH3 phosphorylation may be a single-site phosphorylation event in apple or at least that its Serine 361 plays a crucial role in anthocyanin biosynthesis (Figs 5D–5H, 6 and 7).

### MdHXK1 protein kinase stabilizes MdbHLH3 to regulate the expression of anthocyanin biosynthesis genes

As is well known, the MYB-bHLH-WDR (MBW) complex plays an important role in regulating anthocyanin and proanthocyanidin biosynthesis. In apple, MdbHLH3 physically interacts with MdMYB1 and specifically binds to the promoters of anthocyanin structural genes, such as *MdDFR* and *MdUFGT*, to promote anthocyanin accumulation [32]. Moreover, MdbHLH3 interacts with MdMYB9 and MdMYB11 to regulate the JA-induced biosynthesis of anthocyanin and proanthocyanidin [58]. In the present study, MdbHLH3 promoted anthocyanin accumulation in apple calli and apple fruits (Figs 6 and 7). In addition, MdbHLH3 also promotes malate accumulation in the vacuole by indirectly regulating the vacuolar transport system in apple [59]. Similarly, the increase of malate content in *35S*::*MdHXK1*-overexpressing apple calli accumulated more malate than the WT control (S11 Fig), possibly due to the MdHXK1-mediated stabilization of MdbHLH3.

**Fig 8:**
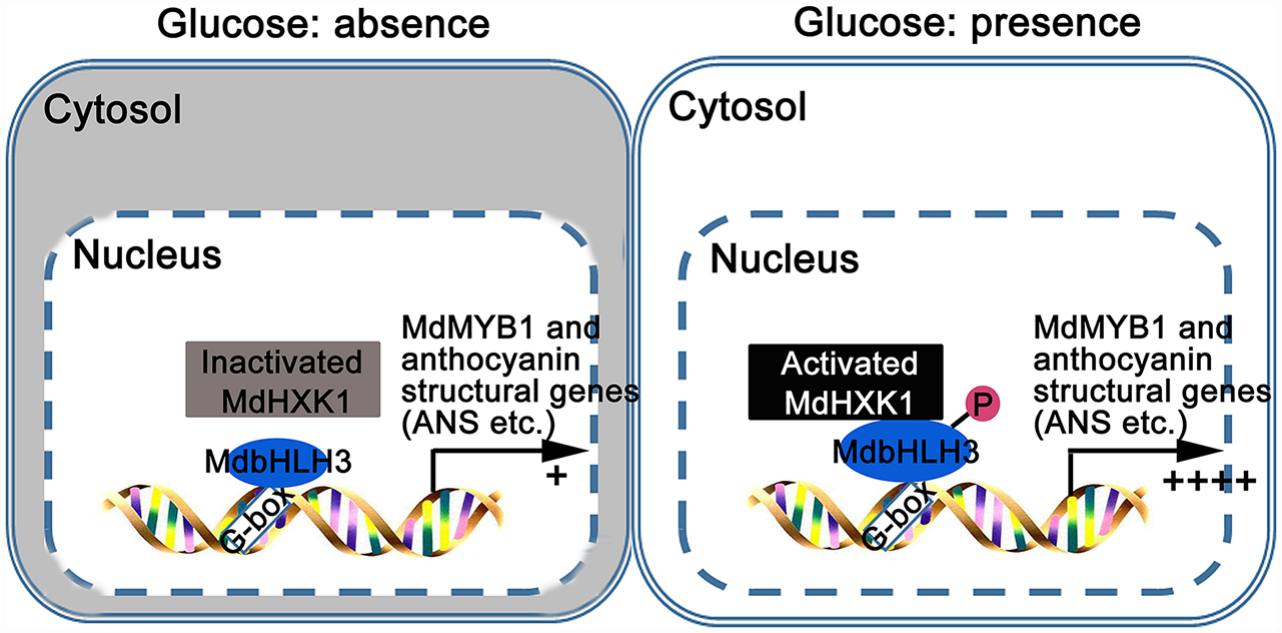
Model demonstrating that MdHXK1 protein kinase stabilizes MdbHLH3 via phosphorylation to modulate anthocyanin accumulation in response to glucose in apple.

The glucose supply promotes anthocyanin biosynthesis and organ coloration in different plant species, such as *Arabidopsis*, grape, and *Paeonia suffruticosa* [36,37,40]. However, the mechanism underlying the glucose signaling-mediated regulation of *MYB* TFs, *WDR* and anthocyanin structural genes remains unclear [37,40]. Here, a working model is proposed to illuminate how glucose regulates anthocyanin accumulation in apple (Fig 8). Under glucose deprivation conditions, the kinase activity of MdHXK1 is inactivated and fails to phosphorylate MdbHLH3 protein (Fig 3A and 3C). As a result, a small amount of MdbHLH3 protein binds to the promoters of anthocyanin structural genes, leading to reduced anthocyanin accumulation (Figs 6 and 7). When exposed to glucose, the kinase activity of MdHXK1 is activated, and then, MdHXK1 phosphorylates and stabilizes the MdbHLH3 protein, which further regulates the expression of the anthocyanin biosynthetic genes and the regulatory *MYB* genes (Figs 4A, 4B, 5 and 6B), ultimately enhancing anthocyanin biosynthesis in apple (Figs 6A, 6C, 7A and 7C). In addition, ectopic expression of the apple *MdHXK1* gene also increased anthocyanin accumulation in the transgenic *Arabidopsis* (S12 Fig), suggesting that the mechanism by which HXK1 controls anthocyanin accumulation in response to glucose is conserved in different species.

In summary, the current study provides new insights into the molecular mechanism of MdHXK1 stabilization of the MdbHLH3 protein, which occurs via phosphorylation, thereby promoting the accumulation of anthocyanins in plant cells in response to glucose signals. Because color is one of the most eye-catching traits for fresh fruits and ornamental plants [60,61], there is considerable interest for the organ coloration in the breeding programs for these economically important plants. Taken together, the regulatory mechanism uncovered in the present study is also useful for the development of novel biotechnological strategies for improving the quality of apple fruit and other horticultural crops.

## Materials and Methods

### Plant materials and growth conditions

The *in vitro* shoot cultures of apple were obtained from detoxified buds of ‘Gala’ apples. They were maintained at 25°C under long-day conditions (16 h light/8 h dark) on Murashige and Skoog (MS) medium supplemented with 0.8 mg L^-1^ 6-BA and 0.2 mg L^-1^ IAA and subcultured at a 4-week interval before being used for further studies.

The apple calli used in this study were induced from the young embryos of the ‘Orin’ apple (*Malus domestica* Borkh.). The calli were grown on MS medium containing 0.5 mg of L-1 indole-3-acetic acid (IAA) and 1.5 mg of L-1 6-benzylaminopurine (6-BA) at 25°C in the dark. The apple calli were subcultured three times at 15-day intervals before being used for genetic transformation and in other assays. Additionally, all the apple calli were suffered from a dark (24 hours dark)-induced glucose starvation before being treated with exogenous glucose in this study, unless noted otherwise.

The apple fruits used for the injection of viral vectors were collected from mature trees of the cultivar ‘Red Delicious’ that were grown in a commercial orchard near Tai-An City. Fruits were bagged at 35 DAB (days after blooming); the bagged fruits were harvested at 140 DAB and de-bagged before injection.

The present study used the *Arabidopsis* (*Arabidopsis thaliana*) ecotype ‘Columbia,’ the *MdHXK1* overexpression line *MdHXK1-OVX1*, the glucose-insensitive mutant *gin2*, and the function-complementary line *MdHXK1-R1* (overexpression of *MdHXK1* in a *gin2* mutant background). Seeds were surface sterilized with 70% (v/v) ethanol and sown on 0.8% (w/v) agar plates containing half-strength MS medium and different glucose concentrations. The seeds were stratified for three days at 4°C and transferred into constant light (100 μmol m^2^ s^-1^) at 20°C for 2 weeks of growth. Before being used for exogenous glucose treatment, 2-weeks-old *Arabidopsis* plants were suffered from a dark (24 hours dark)-induced glucose starvation.

### Construction of the expression vectors and genetic transformation

To construct *MdHXK1* and *MdbHLH3* sense overexpressing and antisense suppressing vectors, the full-length cDNA of *MdHXK1* and *MdbHLH3*, a specific fragment of *MdHXK1* and a conserved fragment of *MdbHLH3* were isolated from ‘Gala’ apple using RT-PCR. Furthermore, truncated sense overexpression vectors, including *MdHXK1*^*35-242aa*^, *MdHXK1*^*245-491aa*^, *MdHXK1*^*1-21aa+35-242aa*^ and *MdHXK1*^*1-21aa+245-491aa*^, were also isolated from ‘Gala’ apple using RT-PCR. All of the cDNA were digested with EcoRI/BamHI and cloned into the pRI plant transformation vector downstream of the CaMV *35S* promoter. All of the primers used in this study are listed in S3 Text.

In addition, two point mutants of *MdHXK1*, namely, G104D (altering Glycine to Aspartate at position 104) and S177A (altering Serine to Alanine at position 177), and the *MdbHLH3* point mutant Ser^361A^ (mutation of Serine to Alanine at position 361), were obtained using site-directed mutagenesis methods. The resulting cDNA were digested with EcoRI/BamHI and cloned into the pRI plant transformation vector downstream of the CaMV *35S* promoter. The primers used in this study are listed in S3 Text.

In addition, the full-length cDNA of *MdHXK1* and *MdbHLH3* were also cloned into the PRI plant transformation vector with a Myc tag downstream of the CaMV *35S* promoter, and subsequently, the recombined expression vectors *MdHXK1-Myc* and *MdbHLH3-Myc* were used for genetic transformation.

For apple calli transformation, the constructs, including *35S*::*MdHXK1*, *35S*::*MdbHLH3*, *35S*::*MdbHLH3*^*S361A*^, *35S*::*MdHXK1*+*35S*::*MdbHLH3*, *35S*::*MdHXK1*+*35S*::*MdbHLH3*^*S361A*^, *35S*::*MdHXK1*+*35S*::*antiMdbHLH3*, *35S*::*antiMdHXK1*+*35S*::*MdbHLH3*, *35S*::*antiMdHXK1*+*35S*::*MdbHLH3*^*S361A*^, *35S*::*antiMdHXK1*+*35S*::*antiMdbHLH3*, and *35S*::*MdbHLH3-Myc*, were introduced into ‘Orin’ apple calli using an *Agrobacterium*-mediated method as described by Hu *et al*. [59].

For *Arabidopsis* transformation, the *35S*::*MdHXK1* vector plasmid was introduced into WT (Col-0) and the glucose-insensitive mutant *gin2* via the *Agrobacterium* strain GV3101 using a floral dip method [59]. The seeds of the transgenic plants were individually harvested and subsequently selfed. Homozygous transgenic lines were used for further investigation.

### RNA extraction and quantitative RT-PCR assays

RNA extraction and quantitative RT-PCR (qRT-PCR) assays were performed with the methods described by Hu *et al*. [59]. All of the primers used for qRT-PCR are listed in S3 Text.

### Protein extraction and Western blotting

Protein extraction and Western blotting assays were conducted as described by Hu *et al*. [59]. The monoclonal antibody of anti-MdHXK1, anti-MdbHLH3^S361^ (specifically against the MdbHLH3 phosphorylation site at residue 361) and anti-GST antibody were prepared by the Abmart Company (Shanghai, China).

### Yeast two-hybrid assays

Yeast two-hybrid assays were performed using the Matchmaker GAL4-based two-hybrid system (Clontech, Palo Alto, CA, USA). Full-length cDNA and truncated mutants of MdHXK1, including MdHXK1^1-245 aa^ and MdHXK1^245-498aa^, were inserted into the pGBT9 vector. The associated yeast two-hybrid vectors of MdbHLH3, which were inserted into vector pGAD424, are detailed in Xie *et al*. [32]. All of the constructs were transformed into yeast strain AH109 using a lithium acetate method. Yeast cells were cultured on minimal medium -Leu/-Trp according to the manufacturer’s instructions. Transformed colonies were plated onto minimal medium -Leu/-Trp/-His/-Ade with or without β-galactosidase to test for possible interactions.

### Co-immunoprecipitation (Co-IP) procedures

The WT and *35S*::*MdbHLH3-GFP* transgenic apple calli were treated with 50 μM MG132 for 16 h to stabilize the MdbHLH3-GFP and MdHXK1 proteins. The Co-IP was carried out as described by Oh *et al*. [62]. The eluted samples were immunoblotted using anti-GFP and anti-MdHXK1 antibodies.

### GST pull-down assays

For the GST pull-down assays, full-length cDNA of *MdbHLH3* were inserted into the pGEX-4T-1 vector, whereas that of *MdHXK1* was inserted into pET-32a. All of the recombinant proteins were used to perform GST pull-down assays as described by Oh *et al*. [62].

### Identification of phosphorylation sites using LC-MS/MS

MdbHLH3 proteins were immunoprecipitated with anti-MdbHLH3 antibody-conjugated agarose beads and then separated on an SDS-PAGE gel and stained with Coomassie brilliant blue (CBB). The band containing phosphorylated MdbHLH3 protein was cut from the stained SDS-PAGE gel. The protein digestion, phosphopeptide enrichment, mass spectrometry data acquisition, data analysis, and label-free quantitation were carried out as described by Wang *et al*. [63].

### Detection of phosphoproteins

The *MdbHLH3-Myc* transgenic apple calli were pre-incubated in MS medium plus 6% glucose with or without 5 U of calf intestine alkaline phosphatase (CIP) for 1 and 3 h. Subsequently, proteins extraction was performed for Western blotting assays with an anti-Myc antibody. Actin served as a protein-loading control.

### *In vitro* kinase assay

A total of 0.2 μg of recombinant His-tagged protein kinase MdHXK1 and 1 μg of MdbHLH3^S361A^-GST and normal MdbHLH3-GST proteins were incubated in 25 μL of reaction buffer [20 mM Tris (pH 7.5), 5 mM MgCl_2_, 10 mM NaCl and 2 mM DTT] with 100 μM ATP and [λ-^32^P]ATP (0.2 mCi per reaction) at room temperature for 30 min. Recombinant MdbHLH3^S361A^-GST was served as a negative control in the *in vitro* kinase assay. The phosphorylated proteins were visualized using autoradiography after separation on a 12% SDS-PAGE gel.

### Construction of the viral vectors and transient expression in apple fruits

To construct antisense expression viral vectors, a specific fragment of *MdHXK1* and a conserved fragment of *MdbHLH3* were amplified with RT-PCR using apple fruit cDNA as the template. The resulting products were cloned into the tobacco rattle virus (TRV) vector in the antisense orientation under the control of the dual *35S* promoter. The vectors were named TRV-MdHXK1 and TRV-MdbHLH3. To generate overexpression viral vectors, full-length cDNA of *MdHXK1* and *MdbHLH3* were inserted into the IL-60 vector under the control of the *35S* promoter. The resulting vectors were named MdHXK1-IL and MdbHLH3-IL.

The antisense expression viral vectors were transformed into *Agrobacterium tumefaciens* strain GV3101 for inoculations. Fruit infiltrations were performed as previously described [59]. The injected apple fruits were kept in the dark at room temperature for two days and subsequently placed in the highlight at 10°C for one week. The peel of the injected part was then harvested for gene expression analysis and anthocyanin content determination.

### Analysis of glucose phosphorylation activity

Glucose phosphorylation activity was measured using an enzyme-linked assay according to Schaffer and Petreikov [64]. The assays contained a total volume of 1 mL of 30 mM HEPES-NaOH, pH 7.5, 2 mM MgCl_2_, 0.6 mM EDTA, 9 mM KCl, 1 mM NAD, 1 mM ATP, and 1 unit of NAD-dependent glucose-6-phosphate dehydrogenase (G6PDH). To assay glucose phosphorylation, 25 μL of the desalted extract was added to start the reaction under 25°C. Reduction of NAD within 5 min was monitored by the increase in absorption at 340 nm. Activity was calculated in terms of μmol of NAD reduced per minute.

### Transient expression in protoplasts of apple calli cells and fluorescence detection

Protoplasts isolated apple calli cells were prepared and transformed as described by Sheen [64]. The fluorescence in transformed cells was detected with a confocal laser scanning microscope (Zeiss LSM 510 META), with excitation wavelengths of 488 nm and 543 nm using an argon laser and an emission wavelength of 505–530 nm and over 560 nm using a BP filter or excitation wavelengths of 458 nm and 514 nm using an argon laser, and an emission wavelength of 475–525 nm and 530–600 nm using a BP filter. A total of 20–30 cells were imaged for each experiment. Co-expressed proteins in the same protoplasts of apple calli cells were detected in the same Pinhole.

### Determination of the total anthocyanin content

The total anthocyanins were extracted using a methanol-HCl method and detected as described by Hu *et al*. [59].

### Statistical analysis

Samples were analyzed in triplicates, and the data are expressed as the mean ± standard deviation unless noted otherwise. Statistical significance was determined using Student’s *t*-test. A difference at P≤0.01 was considered significant, and P≤0.001 was considered extremely significant.

## Supporting Information

S1 Fig Glucose promotes anthocyanin accumulation in an HXK-dependent manner in apple. **(A)** Different concentration of glucose was tested for their ability to induce anthocyanin accumulation in *in vitro* shoot cultures of the ‘Gala’ apple cultivar. The shoot cultures of apple were plated on Murashige and Skoog (MS) agar containing 0.6 mg L^-1^ 6-BA and 0.2 mg L^-1^ IAA plus different concentration of glucose (contains 1%, 2%, 3% and 6%) as indicated. Anthocyanin accumulation in apple leaves was measured after 7 days of growth under 17°C low temperature induction and continuous light. **(B)** Anthocyanin content of apple leaves in **(A)**. **(C)** The phenotype as indicated by a red color for anthocyanin accumulation in *in vitro* shoot cultures of the ‘Gala’ apple cultivar treated with 6% glucose and 6% mannitol or 6% glucose plus glucosamine. **(D)** The anthocyanin content of apple shoot cultures in **(C)**. The data are shown as the mean ± SE, which were analyzed based on more than 9 replicates. Statistical significance was determined using Student’s *t*-test in apple shoot cultures. n.s., p > 0.01; **p < 0.001.
(TIF)

S2 Fig Amino acid sequence alignment of apple MdHXK1 and *Arabidopsis* AtHXK1 proteins. The conserved amino acid residues were labeled with black boxes. The alignment of sequences was generated using a ‘‘multiple sequence alignment” method with DNAMAN software.
(TIF)

S3 Fig Glucose modulates anthocyanin accumulation by the signaling and catalytic pathway under 1% glucose condition. **(A)** WT, *35S*::*MdHXK1* (WT background), *35S*::*MdHXK1*^*G104D*^ (WT background), and *35S*::*MdHXK1*^*S177A*^ (WT background) transgenic apple calli showed anthocyanin accumulation phenotype on MS agar media containing 1% glucose. The apple calli were placed at 10°C under long-day conditions (16 h light/8 h dark) for 10 days. **(B)** and **(C)** Anthocyanin content **(B)** and glucose phosphorylation activity **(C)** in WT and transgenic apple calli in **(A)**. In **(B)** and **(C)**, data are shown as mean±SE, which were analyzed based on more than 9 replicates. Statistical significance was determined using Student’s *t* test in different apple calli lines. n.s., P > 0.01; *P < 0.01; **P < 0.001.
(TIF)

S4 Fig Immunoblotting assays and analysis of MdHXK1-binding proteins with Co-IP and the domain structures of the MdHXK1 protein. **(A)** Western blotting assay of MdHXK1 protein abundance by using Myc antibody in wild type (WT) and *35S*::*MdHXK1-Myc* transgenic apple calli. The ACTIN was served as a protein-loading control. **(B)** Co-IP assay of MdHXK1-interacting proteins in MdHXK1-Myc transgenic apple calli. Co-immunoprecipitation assay was performed by using monoclonal Anti-Myc antibody to screen the MdHXK1-binding proteins. The resultant IPed proteins was detected by coomassie blue staining. **(C)** Schematic diagram of the domain structures of MdHXK1. The domain prediction was performed on the website http://smart.embl-heidelberg.de/. The pink rectangle indicates the signal peptide, while the gray rectangle shows the hexokinase_1 and hexokinase_2 domains respectively. The numbers below domains indicated the predicted starting and ending numbers of amino acid.
(TIF)

S5 Fig Western blotting assay of the MdbHLH3 protein with anti-Myc and anti-MdbHLH3^S361^ antibodies. **(A)** Western blotting assay of MdbHLH3 protein abundance by using anti-Myc antibody in wild type (WT) and *35S*::*MdbHLH3-Myc* transgenic apple calli. The ACTIN was served as a protein-loading control. **(B)** Western blotting assay of the specificity of the anti-MdbHLH3^S361^ antibody. The *MdbHLH3-Myc* transgenic apple calli was pre-incubated in MS medium plus 6% glucose for 3 hours. Subsequently, the proteins extraction was used for Western blotting assays with an antibody of MdbHLH3^S361^ phosphorylation site. **(C)** Glucose induced the phosphorylation of MdbHLH3 protein and was abolished by CIP in WT apple plants. The apple plants were pre-incubated in MS medium plus glucose (0 or 6%) and 5 U of CIP for 1 or 3 hours. Subsequently, the proteins extraction was used for Western blotting assays with an antibody of MdbHLH3^S361^ phosphorylation site.
(TIF)

S6 Fig The transcript and protein level of MdHXK1 in wild-type (WT) and *35S*::*antiMdHXK1* transgenic apple calli. **(A)** Relative expression level of *MdHXK1* in WT and *35S*::*antiMdHXK1* transgenic apple calli. Data are shown as mean±SE, which were analyzed based on more than 9 replicates. Statistical significance was determined using Student’s *t* test in different apple calli lines. *P < 0.01. **(B)** The protein abundance of MdHXK1 in WT and *35S*::*antiMdHXK1* transgenic apple calli.
(TIF)

S7 Fig MdHXK1 *in vitro* phosphorylates MdbHLH3 but not MdbHLH3^S^^361A^. The kinase assay was initiated by adding radiolabeled ATP to the mixture of MdHXK1-His kinase and MdbHLH3-GST (or MdbHLH3^S361A^-GST). The phosphorylated MdbHLH3-GST protein were detected with anti-MdbHLH3^S361^ antibody. Note: MdbHLH3-GST-P represent the phosphorylated MdbHLH3-GST protein.
(TIF)

S8 Fig MdHXK1 *in vivo* kinase activity stabilizes the MdbHLH3 protein. **(A-C)** MdHXK1 *in vivo* kinase activity stabilizes MdbHLH3 protein. The isolated total proteins from *35S*::*MdHXK1* **(A)**, WT **(B)** and *35S*::*antiMdHXK1* **(C)** apple calli were treated with 20 μg /ml cycloheximide for 0, 1, 3 and 6 h. The degradation of MdbHLH3 protein was followed by western blotting with anti-MdbHLH3 antibody. **(D)** The graph shows the quantitation of the western blot data in **(A)**, **(B)**, and **(C)**.
(TIF)

S9 Fig Western blotting assay of MdbHLH3^S^^361A^ protein abundance using a Myc antibody in wild-type (WT) and *35S*::*MdbHLH3* ^*S*^^*361A*^*-Myc* transgenic apple calli. The ACTIN was served as a protein-loading control.
(TIF)

S10 Fig MdHXK1 activates the *MdANR* promoter as detected by GUS assays. **(A)** The effectors and reporter constructs in the binary vectors were introduced into apple calli for GUS activity assays. *pMdANR*::*GUS* transgenic apple calli were transformation with *35S*::*MdHXK1* (MdHXK1-overexpressing vector), empty vector and *35S*::*antiMdHXK1* (MdHXK1-suppressing vector) were grown at 25°C in the dark and stained to detect GUS activity. **(B)** GUS activity in the transgenic apple calli as labeled in **(A)**. The means and standard deviations were calculated from the results of three independent experiments.
(TIF)

S11 Fig Malate content in the WT control as well as the *35S*::*MdHXK1* overexpressing and *35S*::*antiMdHXK1* suppressing transgenic apple calli. (TIF)

S12 Fig The mechanism through which glucose-mediated HXK1 controls anthocyanin accumulation is conserved in different species. **(A)** Glucose-mediated HXK1 promotes anthocyanin biosynthesis in *Arabidopsis*. The WT (Ler), *AtHXK1* mutant *gin2*, *MdHXK1* overexpressor line HXK1-OVX1 and function complementary line HXK1-R1 were grown on one-half-strength MS medium without sugar (Control) or with 6% glucose (w/v), and 6% mannitol (w/v) at 10°C under long-day conditions (16 h light/8 h dark) for 10 days. **(B)** Anthocyanin content of WT and transgenic *arabidopsis* as indicated in **(A)**. **(C)** Amino acid alignment of the conserved Ser^361^ of HXK1 proteins in apple and other species. The conserved serine residue at position 361 were indicated with red pentagram. The alignment of all the sequences was generated using a ‘‘multiple sequence alignment” method with DNAMAN software.
(TIF)

S1 Text Identification of MdHXK1-interacting proteins in co-immunoprecipitation using an LC/MS assay. (XLS)

S2 Text Identification of potential phosphorylation sites in MdbHLH3 with an LC/MS assay. (XLS)

S3 Text List of primers used in this study. (XLS)

S4 Text Map of potential phosphorylation sites in MdbHLH3 with an LC/MS assay. (PDF)

S5 Text Glucose promotes anthocyanin accumulation in an HXK-dependent manner in apple. (DOCX)
