# Supplementary figures and images for: Glucose Sensor MdHXK1 Phosphorylates and Stabilizes MdbHLH3 to Promote Anthocyanin Biosynthesis in Apple
Source: PLoS Genet. 2016 Aug 25;12(8):e1006273. doi: 10.1371/journal.pgen.1006273 (PMC4999241; doi:10.1371/journal.pgen.1006273)

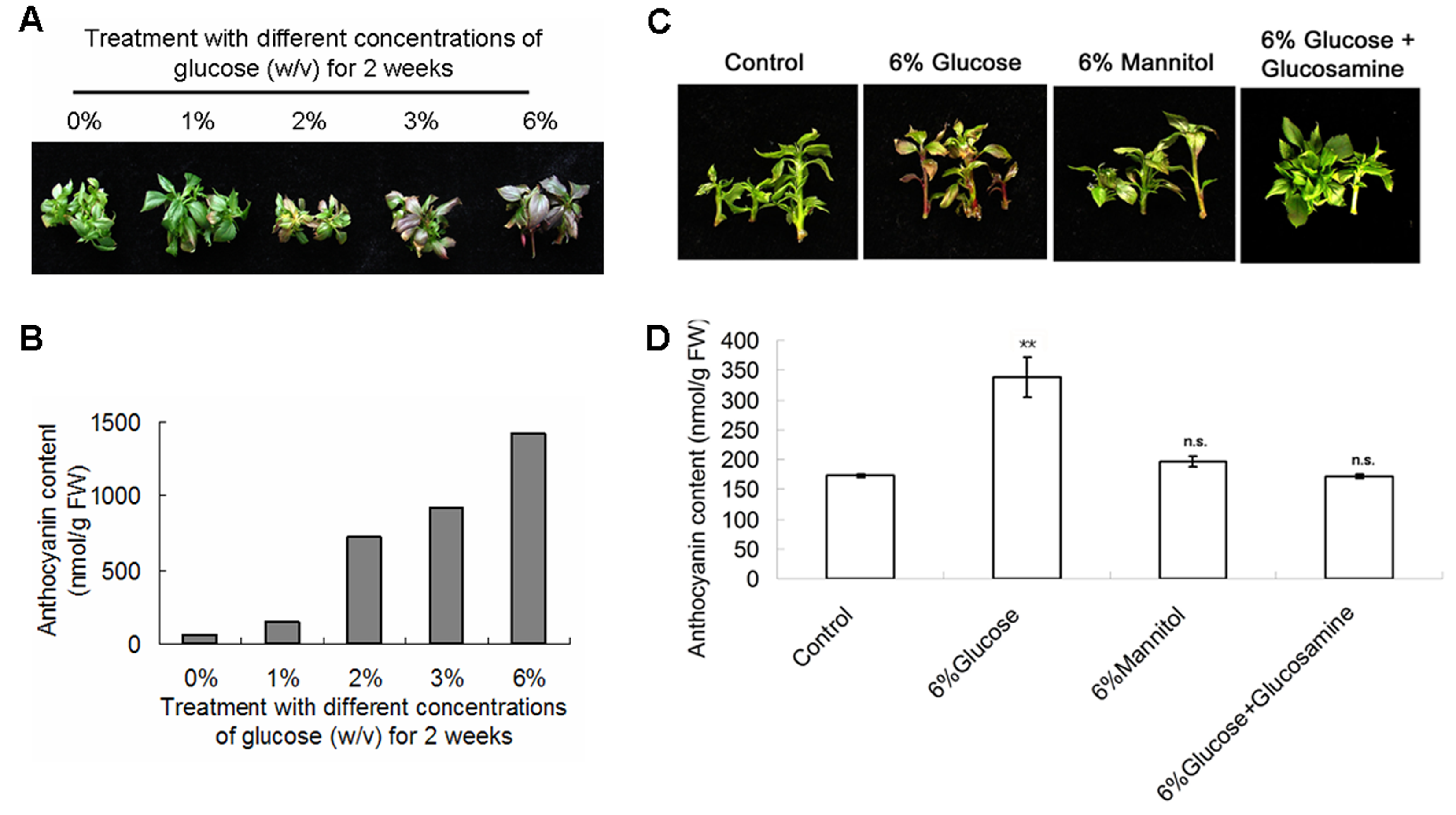

Supplement: S1 Fig — (A) Different concentration of glucose was tested for their ability to induce anthocyanin accumulation in in vitro shoot cultures of the ‘Gala’ apple cultivar. The shoot cultures of apple were plated on Murashige and Skoog (MS) agar containing 0.6 mg L-1 6-BA and 0.2 mg L-1 IAA plus different concentration of glucose (contains 1%, 2%, 3% and 6%) as indicated. Anthocyanin accumulation in apple leaves was measured after 7 days of growth under 17°C low temperature induction and continuous light. (B) Anthocyanin content of apple leaves in (A). (C) The phenotype as indicated by a red color for anthocyanin accumulation in in vitro shoot cultures of the ‘Gala’ apple cultivar treated with 6% glucose and 6% mannitol or 6% glucose plus glucosamine. (D) The anthocyanin content of apple shoot cultures in (C). The data are shown as the mean ± SE, which were analyzed based on more than 9 replicates. Statistical significance was determined using Student’s t-test in apple shoot cultures. n.s., p > 0.01; **p < 0.001. (TIF) [file pgen.1006273.s001.tif]

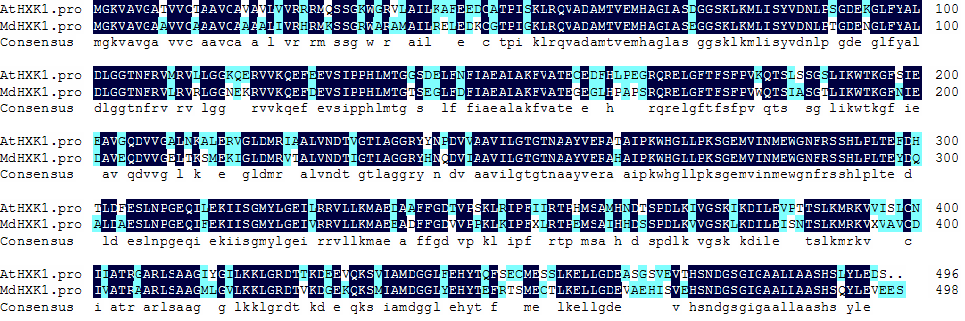

Supplement: S2 Fig — The conserved amino acid residues were labeled with black boxes. The alignment of sequences was generated using a ‘‘multiple sequence alignment” method with DNAMAN software. (TIF) [file pgen.1006273.s002.tif]

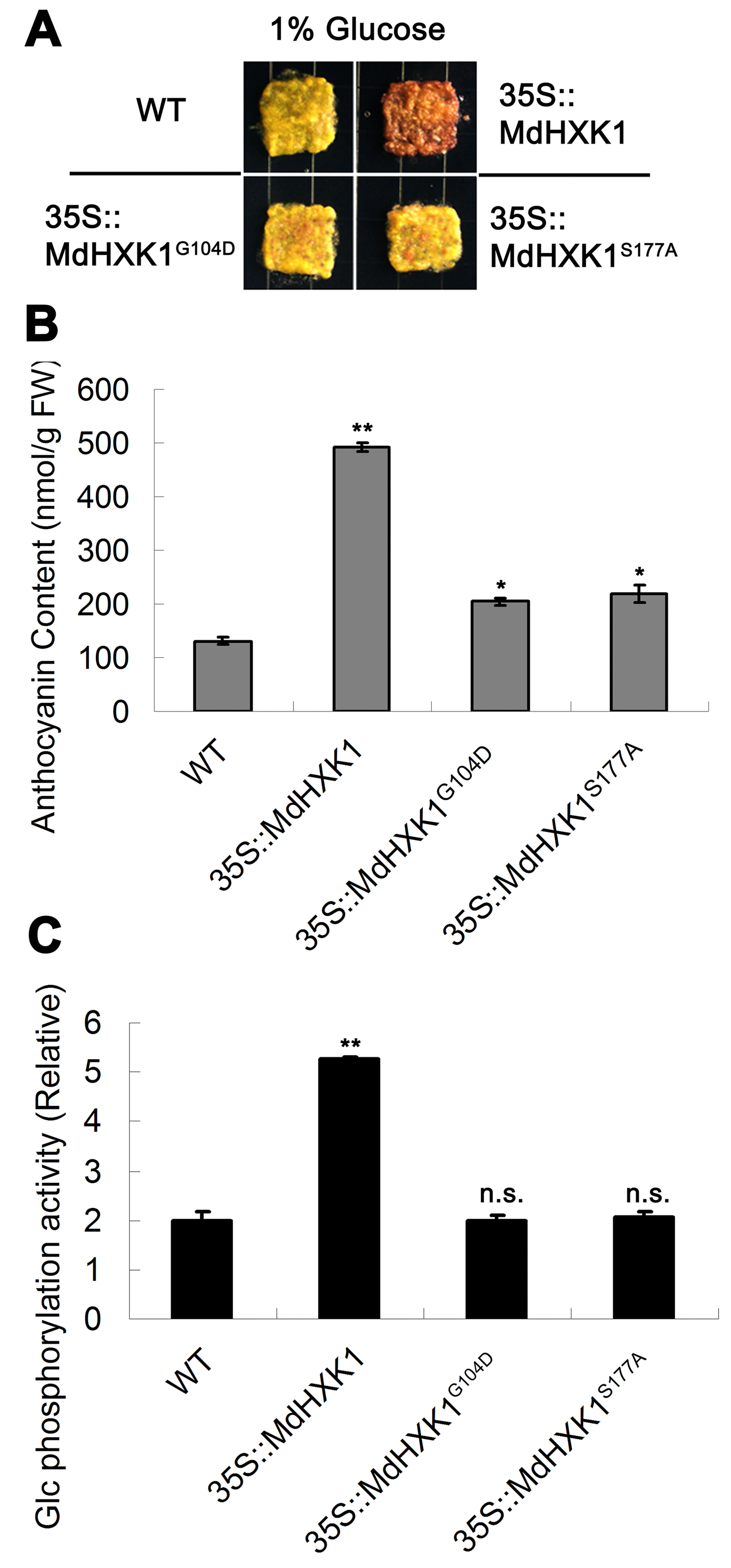

Supplement: S3 Fig — (A) WT, 35S::MdHXK1 (WT background), 35S::MdHXK1G104D (WT background), and 35S::MdHXK1S177A (WT background) transgenic apple calli showed anthocyanin accumulation phenotype on MS agar media containing 1% glucose. The apple calli were placed at 10°C under long-day conditions (16 h light/8 h dark) for 10 days. (B) and (C) Anthocyanin content (B) and glucose phosphorylation activity (C) in WT and transgenic apple calli in (A). In (B) and (C), data are shown as mean±SE, which were analyzed based on more than 9 replicates. Statistical significance was determined using Student’s t test in different apple calli lines. n.s., P > 0.01; *P < 0.01; **P < 0.001. (TIF) [file pgen.1006273.s003.tif]

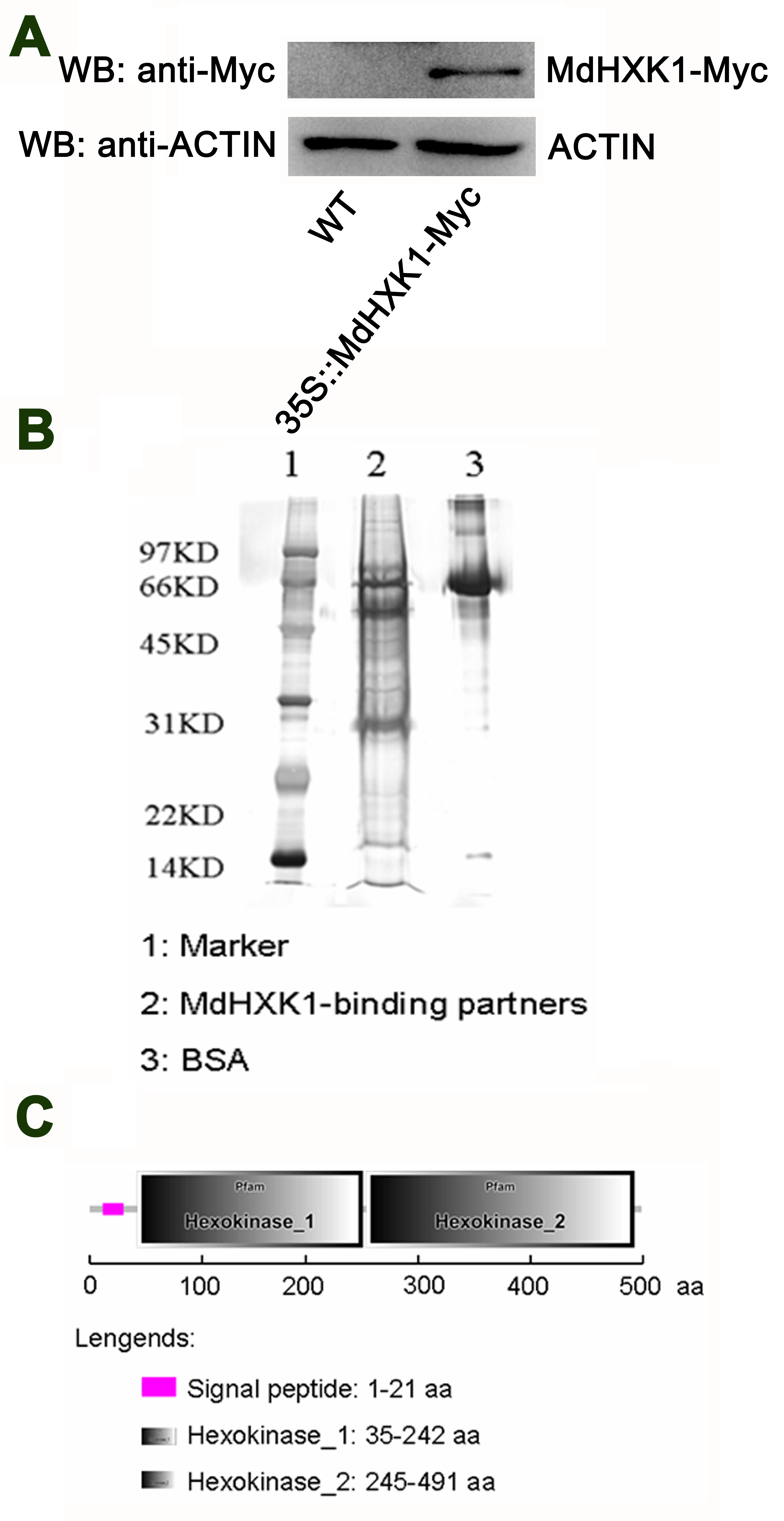

Supplement: S4 Fig — (A) Western blotting assay of MdHXK1 protein abundance by using Myc antibody in wild type (WT) and 35S::MdHXK1-Myc transgenic apple calli. The ACTIN was served as a protein-loading control. (B) Co-IP assay of MdHXK1-interacting proteins in MdHXK1-Myc transgenic apple calli. Co-immunoprecipitation assay was performed by using monoclonal Anti-Myc antibody to screen the MdHXK1-binding proteins. The resultant IPed proteins was detected by coomassie blue staining. (C) Schematic diagram of the domain structures of MdHXK1. The domain prediction was performed on the website http://smart.embl-heidelberg.de/. The pink rectangle indicates the signal peptide, while the gray rectangle shows the hexokinase_1 and hexokinase_2 domains respectively. The numbers below domains indicated the predicted starting and ending numbers of amino acid. (TIF) [file pgen.1006273.s004.tif]

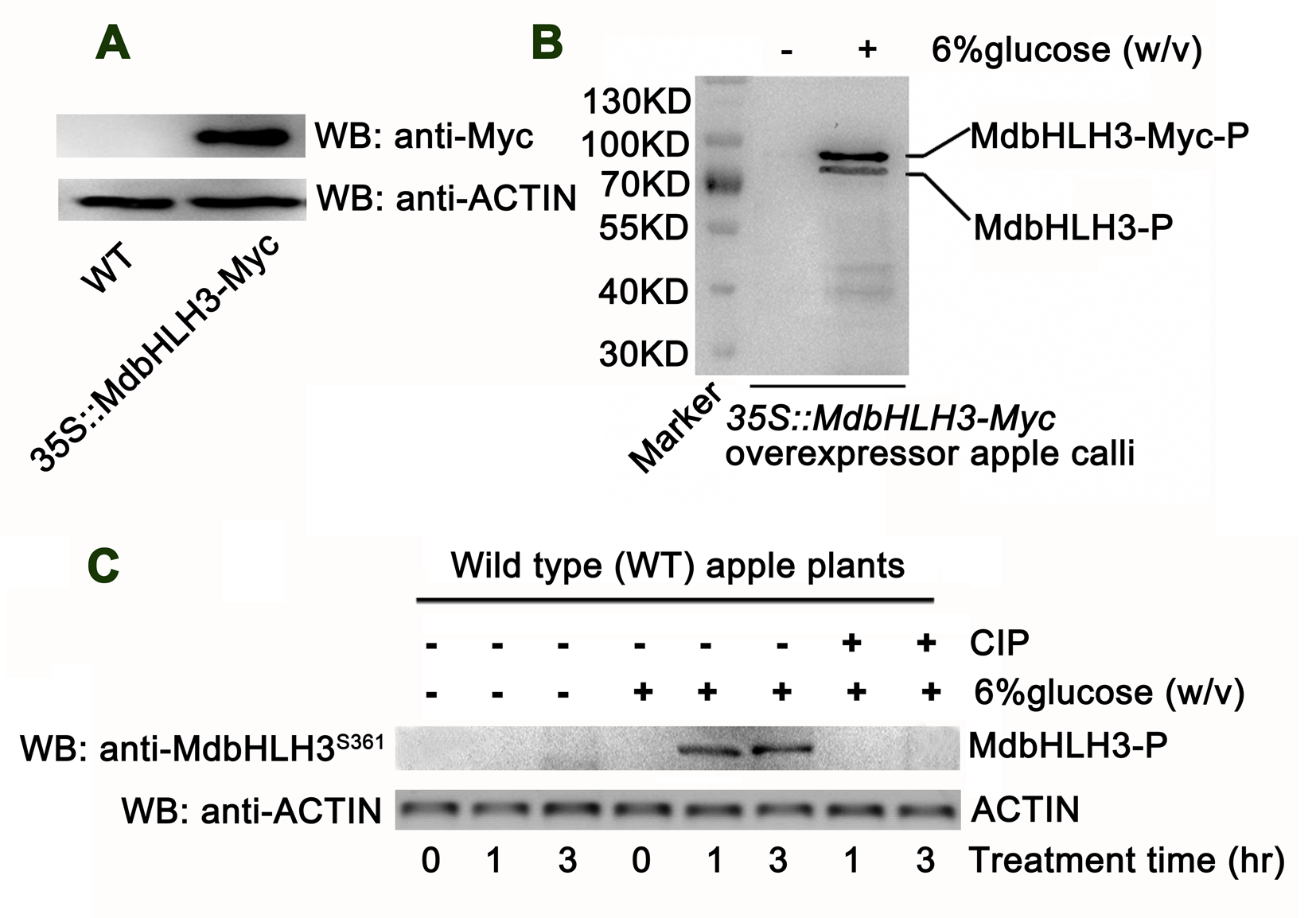

Supplement: S5 Fig — (A) Western blotting assay of MdbHLH3 protein abundance by using anti-Myc antibody in wild type (WT) and 35S::MdbHLH3-Myc transgenic apple calli. The ACTIN was served as a protein-loading control. (B) Western blotting assay of the specificity of the anti-MdbHLH3S361 antibody. The MdbHLH3-Myc transgenic apple calli was pre-incubated in MS medium plus 6% glucose for 3 hours. Subsequently, the proteins extraction was used for Western blotting assays with an antibody of MdbHLH3S361 phosphorylation site. (C) Glucose induced the phosphorylation of MdbHLH3 protein and was abolished by CIP in WT apple plants. The apple plants were pre-incubated in MS medium plus glucose (0 or 6%) and 5 U of CIP for 1 or 3 hours. Subsequently, the proteins extraction was used for Western blotting assays with an antibody of MdbHLH3S361 phosphorylation site. (TIF) [file pgen.1006273.s005.tif]

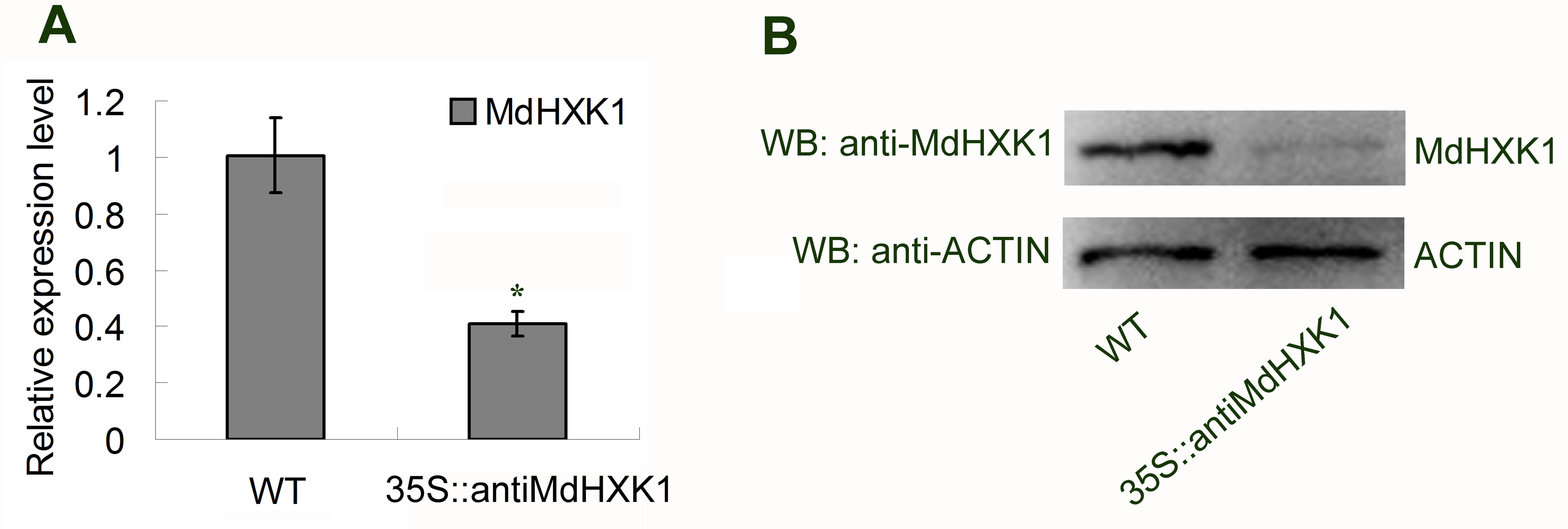

Supplement: S6 Fig — (A) Relative expression level of MdHXK1 in WT and 35S::antiMdHXK1 transgenic apple calli. Data are shown as mean±SE, which were analyzed based on more than 9 replicates. Statistical significance was determined using Student’s t test in different apple calli lines. *P < 0.01. (B) The protein abundance of MdHXK1 in WT and 35S::antiMdHXK1 transgenic apple calli. (TIF) [file pgen.1006273.s006.tif]

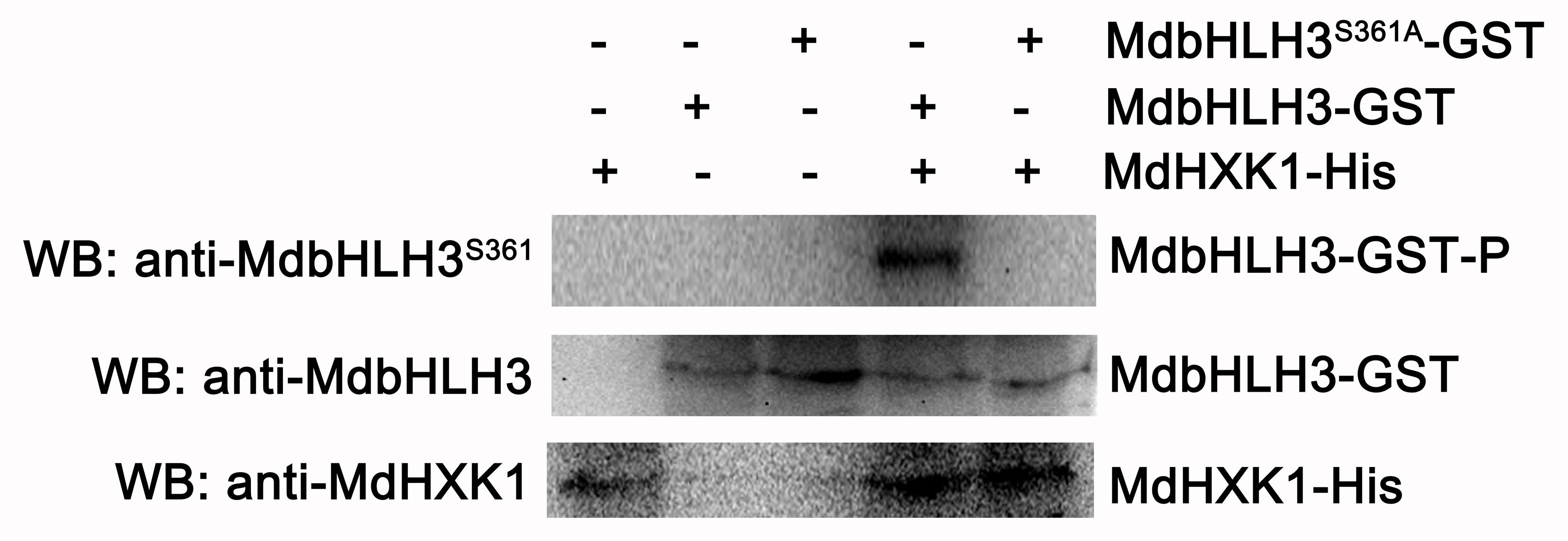

Supplement: S7 Fig — The kinase assay was initiated by adding radiolabeled ATP to the mixture of MdHXK1-His kinase and MdbHLH3-GST (or MdbHLH3S361A-GST). The phosphorylated MdbHLH3-GST protein were detected with anti-MdbHLH3S361 antibody. Note: MdbHLH3-GST-P represent the phosphorylated MdbHLH3-GST protein. (TIF) [file pgen.1006273.s007.tif]

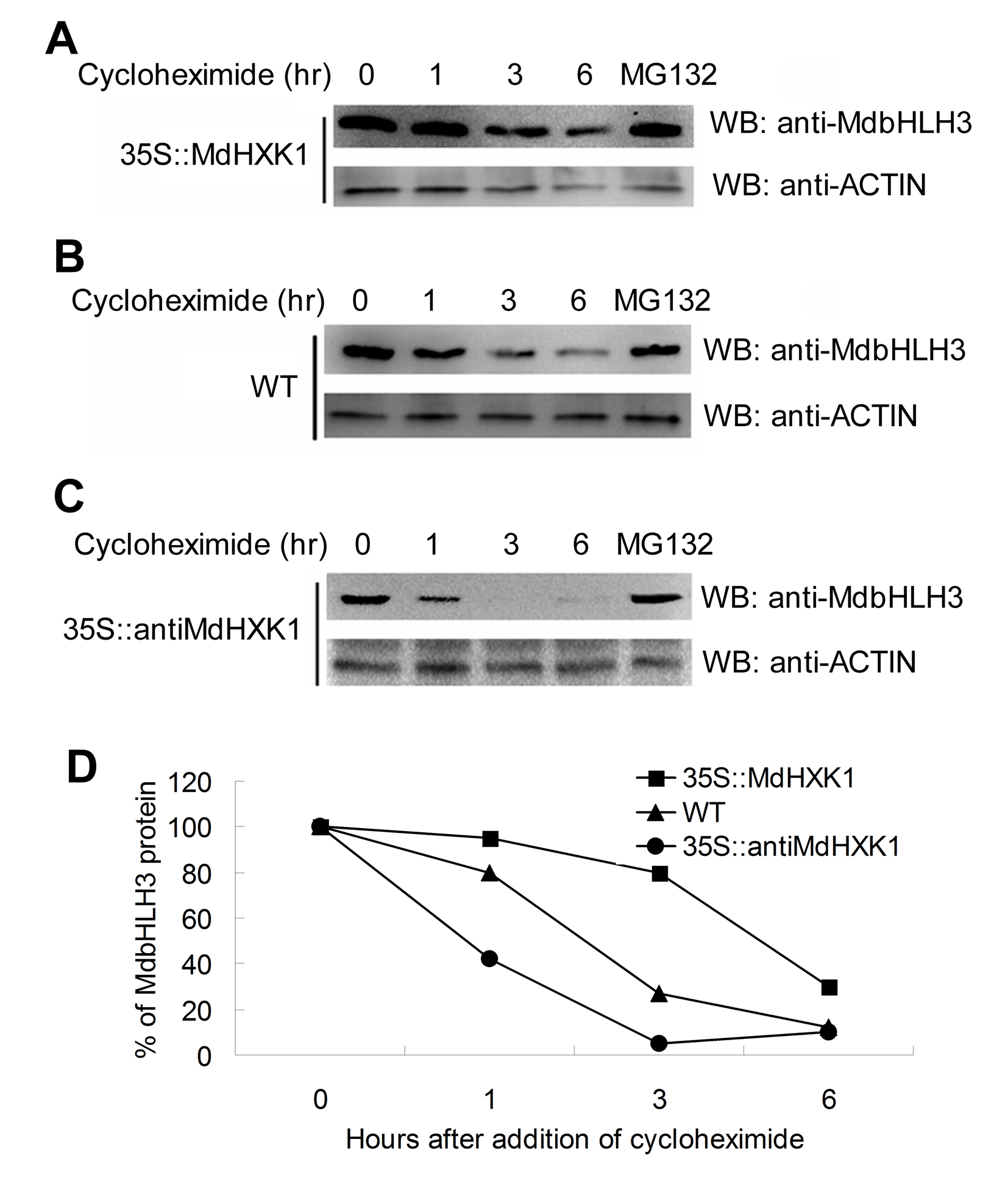

Supplement: S8 Fig — (A-C) MdHXK1 in vivo kinase activity stabilizes MdbHLH3 protein. The isolated total proteins from 35S::MdHXK1 (A), WT (B) and 35S::antiMdHXK1 (C) apple calli were treated with 20 μg /ml cycloheximide for 0, 1, 3 and 6 h. The degradation of MdbHLH3 protein was followed by western blotting with anti-MdbHLH3 antibody. (D) The graph shows the quantitation of the western blot data in (A), (B), and (C). (TIF) [file pgen.1006273.s008.tif]

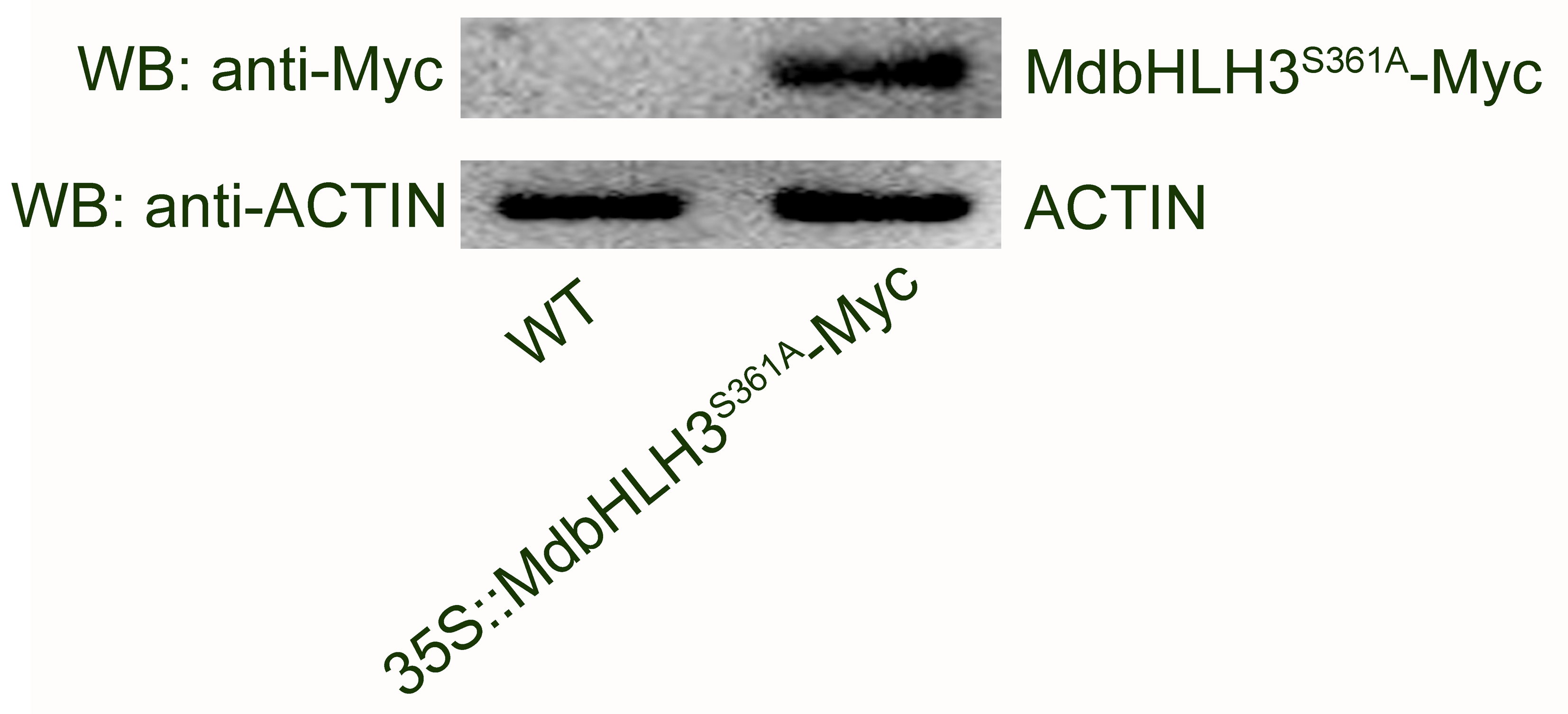

Supplement: S9 Fig — The ACTIN was served as a protein-loading control. (TIF) [file pgen.1006273.s009.tif]

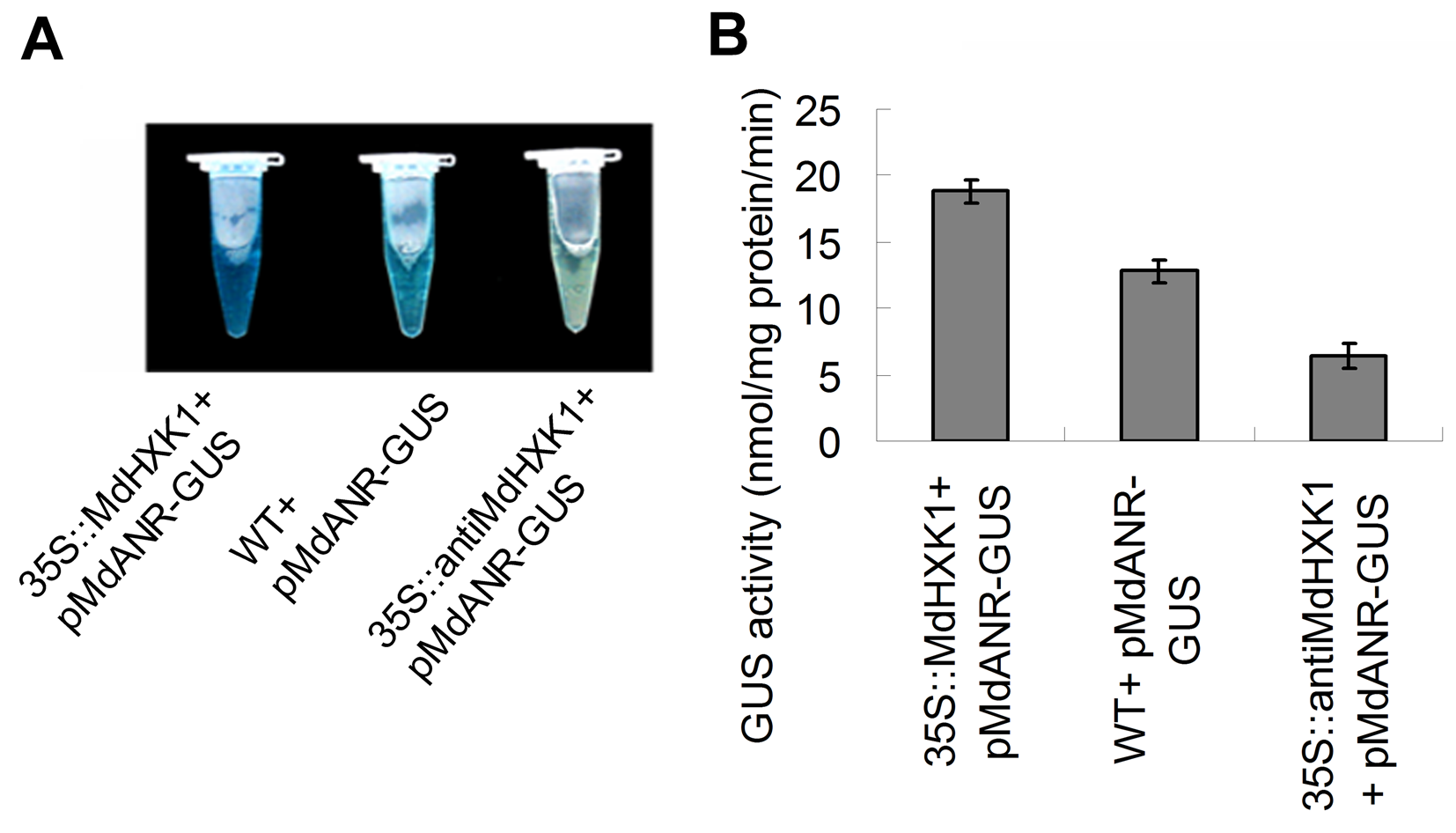

Supplement: S10 Fig — (A) The effectors and reporter constructs in the binary vectors were introduced into apple calli for GUS activity assays. pMdANR::GUS transgenic apple calli were transformation with 35S::MdHXK1 (MdHXK1-overexpressing vector), empty vector and 35S::antiMdHXK1 (MdHXK1-suppressing vector) were grown at 25°C in the dark and stained to detect GUS activity. (B) GUS activity in the transgenic apple calli as labeled in (A). The means and standard deviations were calculated from the results of three independent experiments. (TIF) [file pgen.1006273.s010.tif]

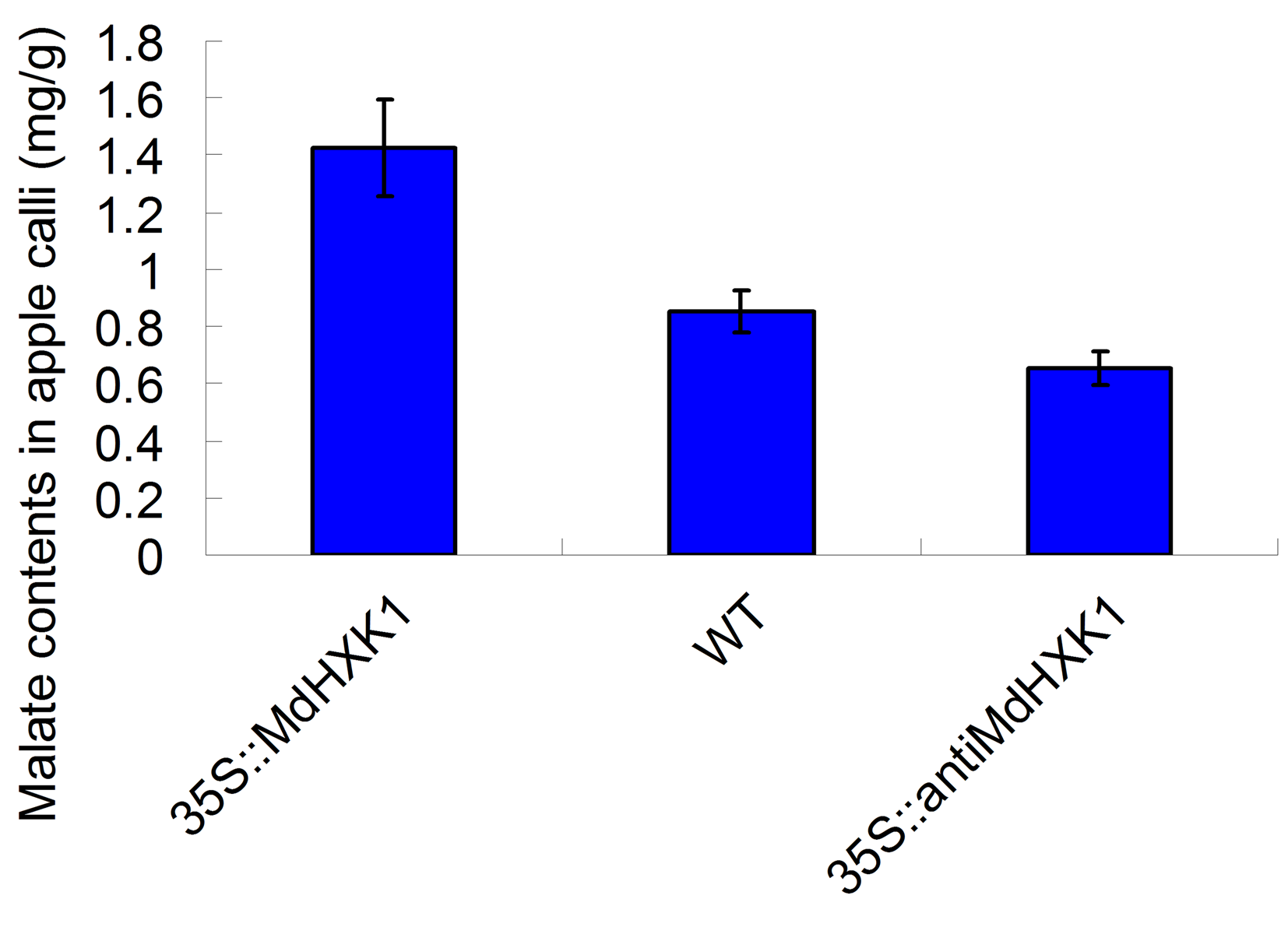

Supplement: S11 Fig — (TIF) [file pgen.1006273.s011.tif]

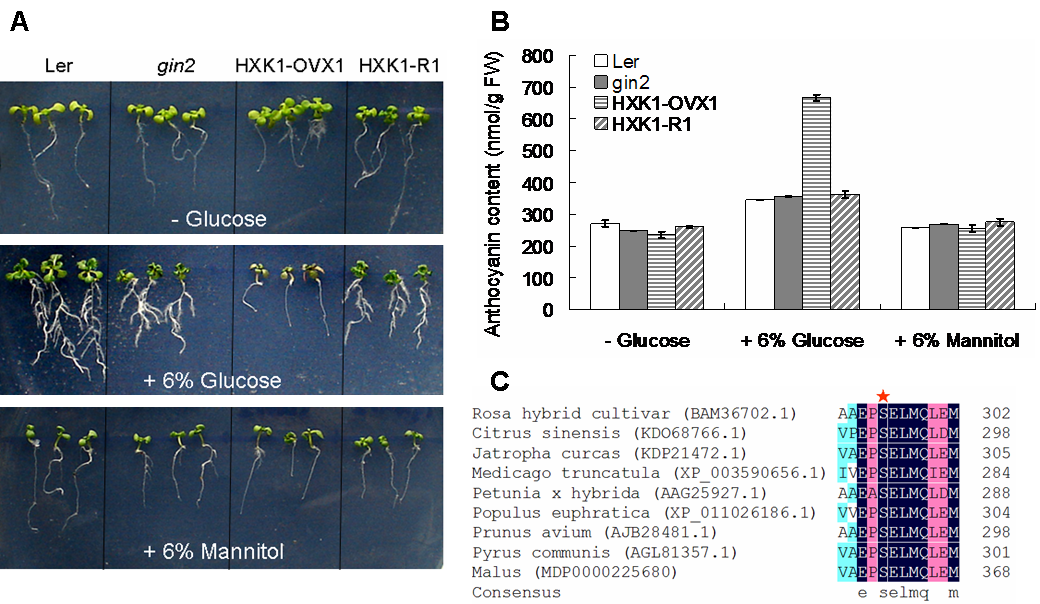

Supplement: S12 Fig — (A) Glucose-mediated HXK1 promotes anthocyanin biosynthesis in Arabidopsis. The WT (Ler), AtHXK1 mutant gin2, MdHXK1 overexpressor line HXK1-OVX1 and function complementary line HXK1-R1 were grown on one-half-strength MS medium without sugar (Control) or with 6% glucose (w/v), and 6% mannitol (w/v) at 10°C under long-day conditions (16 h light/8 h dark) for 10 days. (B) Anthocyanin content of WT and transgenic arabidopsis as indicated in (A). (C) Amino acid alignment of the conserved Ser361 of HXK1 proteins in apple and other species. The conserved serine residue at position 361 were indicated with red pentagram. The alignment of all the sequences was generated using a ‘‘multiple sequence alignment” method with DNAMAN software. (TIF) [file pgen.1006273.s012.tif]

R14065\_1\_Bhlh3\_GST

R14065\_1\_Bhlh3\_GST #17924 RT: 40.20 AV: 1 NL: 1.71E4  
T: FTMS + p NSI d Full ms2 901.18@hcd28.00 [100.00-3700.00]

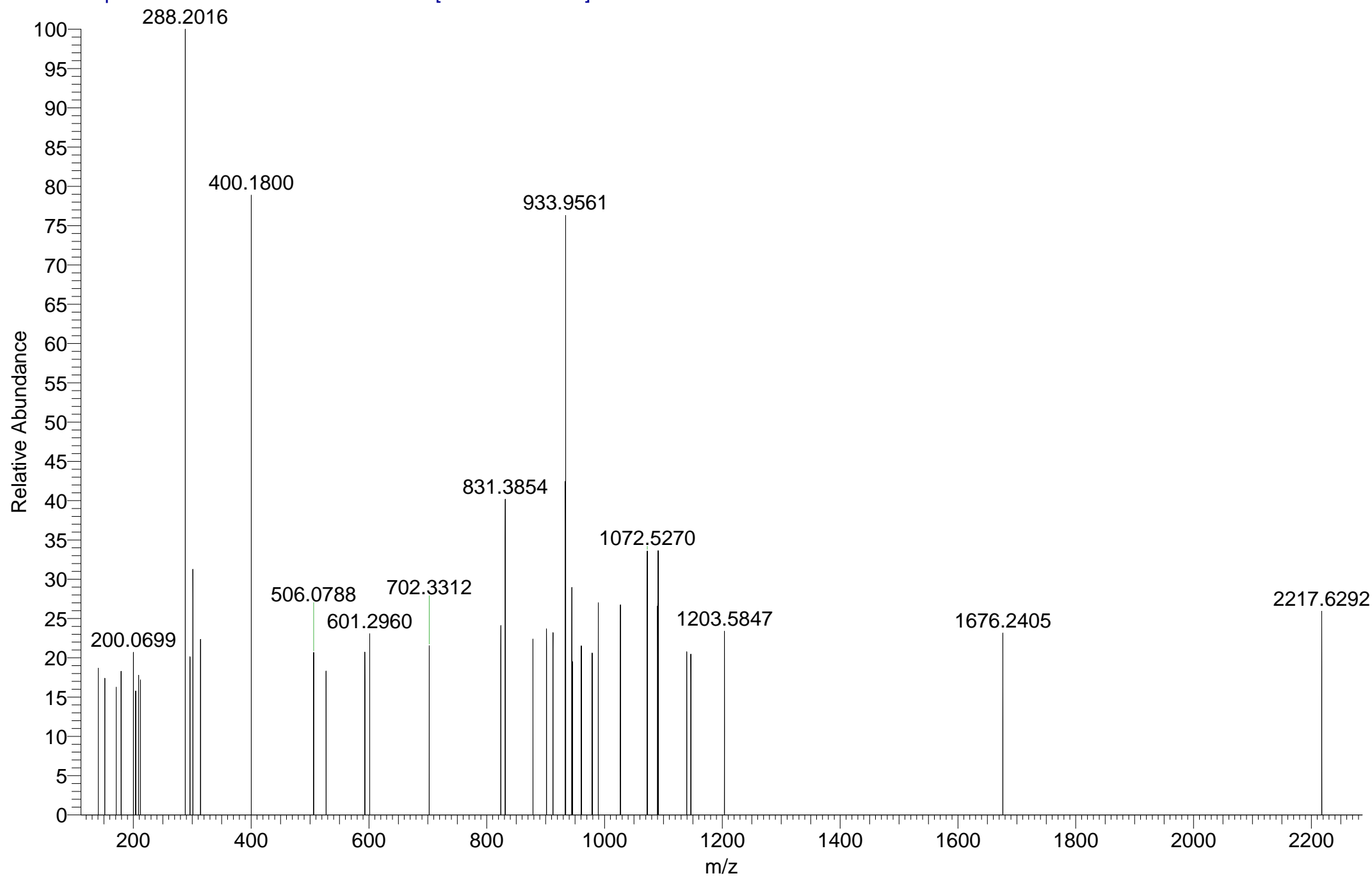

Supplement: S4 Text — (PDF) [file pgen.1006273.s016.pdf]
