# Supplementary material for: Glucose Sensor MdHXK1 Phosphorylates and Stabilizes MdbHLH3 to Promote Anthocyanin Biosynthesis in Apple
Source: PLoS Genet. 2016 Aug 25;12(8):e1006273. doi: 10.1371/journal.pgen.1006273 (PMC4999241; doi:10.1371/journal.pgen.1006273)
Supplement: S5 Text — (DOCX) [file pgen.1006273.s017.docx]

**S5 Text. Glucose promotes anthocyanin accumulation in an HXK-dependent manner in apple**

Previous studies have verified that glucose significantly induces anthocyanin biosynthesis in *Arabidopsis* seedlings (Teng et al., 2005). Similarly, the effect of different concentrations of glucose (0-6%, w/v) on anthocyanin accumulation was tested in *in vitro* shoot cultures of the ‘Gala’ apple cultivar. The result showed that the accumulation of anthocyanins increased with increases in the external glucose concentration, especially with 6% (w/v) glucose (**S1A, 1B Fig**).

Subsequently, the shoot cultures were treated with 6% glucose, and 6% mannitol was used to mimic osmotic stress. The result showed that anthocyanin accumulation was attributed to 6% glucose but not to osmotic stress (**S1C, 1D Fig**). In addition, to investigate whether hexokinase (HXK) is involved in glucose-induced anthocyanin accumulation, the HXK inhibitor glucosamine was used to treat the shoot cultures growing in Murashige and Skoog (MS) medium plus 6% glucose. Interestingly, glucosamine significantly reduced the glucose-induced anthocyanins in apple shoot cultures (**S1C, 1D Fig**). These results indicate that the glucose-induced anthocyanin accumulation depends on the function of HXK in apple.
